# Neddylation of NFATc1 and Runx2 regulates osteoclast–osteoblast balance and represents a dual-action therapeutic target for postmenopausal osteoporosis

**DOI:** 10.1038/s12276-026-01784-2

**Published:** 2026-08-03

**Authors:** Jooseung Lee, Min Young Lee, Hong Seok Kim, Jong-Wan Park, Geon Ho Moon, Jun Bum Park, Hye-Jin Kim, Sung Joon Kim, Yang-Sook Chun

**Affiliations:** 1 Department of Biomedical Sciences, Seoul National University College of Medicine, Seoul, South Korea; 2 Department of Physiology, Seoul National University College of Medicine, Seoul, South Korea; 3 Department of Orthopedic Surgery, Seoul National University Hospital, Seoul National University College of Medicine, Seoul, South Korea; 4 Ischemic/Hypoxic Disease Institute, Seoul National University College of Medicine, Seoul, South Korea; 5 Department of Medicine, Massachusetts General Hospital, Harvard Medical School, Boston, MA USA

**Keywords:** Neddylation, Osteoporosis

## Abstract

Postmenopausal osteoporosis (PMOP) arises from a bone remodeling imbalance in which osteoclast-mediated resorption exceeds osteoblast-driven formation. However, current therapies primarily target osteoclasts without restoring osteoblast function, underscoring the need for mechanism-driven dual-action approaches. Here, we identify neddylation as a post-translational modification that reciprocally regulates osteoclast and osteoblast differentiation. In bone tissue from patients with PMOP, NEDD8 and its activating enzyme NAE1 were significantly upregulated, indicating heightened pathway activity. Mechanistically, c-Cbl neddylates NFATc1 to promote osteoclastogenesis, whereas Rbx1 neddylates Runx2 to suppress osteoblast maturation. Pharmacological blockade with the NAE1 inhibitor MLN4924 counteracted both effects and, in ovariectomized mice, reduced osteoclast activity while preserving osteoblast function, preventing deterioration of bone mass and microarchitecture. In human PMOP bone tissues, elevated NEDD8 and NAE1 expression was associated with increased NFATc1 in osteoclast-lineage cells and reduced Runx2 in osteoblast-lineage cells, suggesting translational relevance. These findings reveal neddylation as a unifying mechanism that coordinates opposing effects on osteoclast and osteoblast master transcription factors and highlight its potential as a mechanism-driven dual-action therapeutic target.

**Figure:**
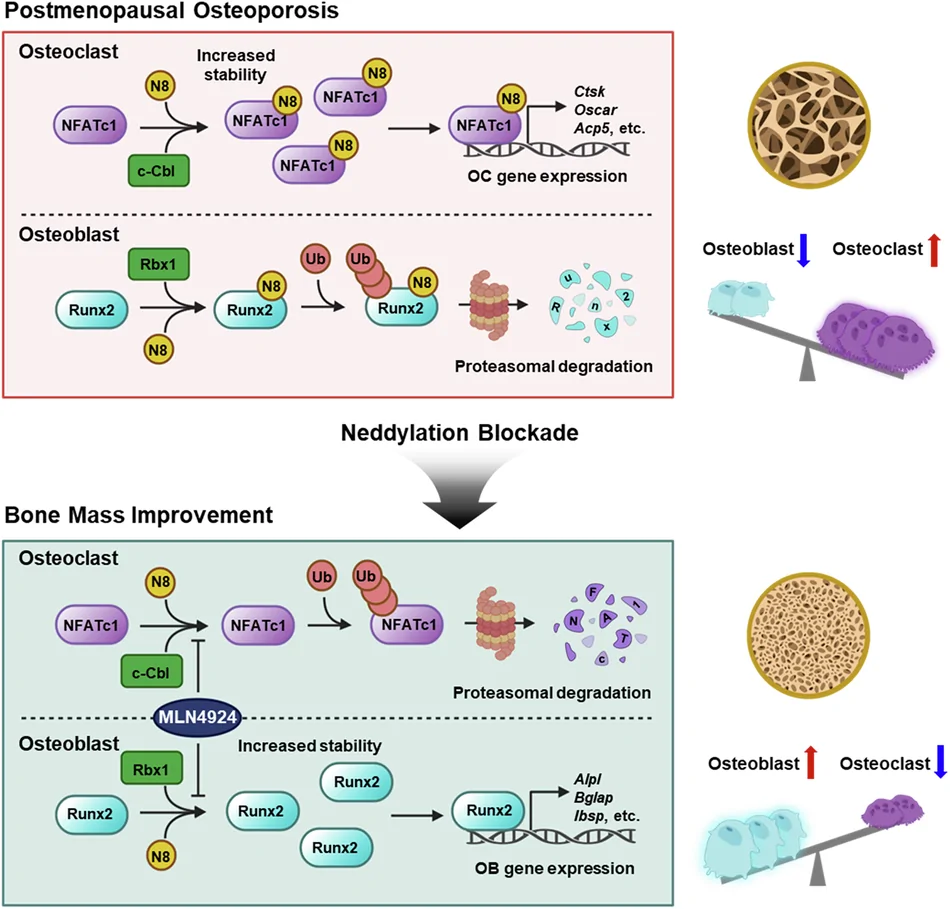

## Neddylation balances osteoclast and osteoblast activity in bones

Postmenopausal osteoporosis (PMOP) poses a significant health challenge, characterized by bone loss and increased fracture risk owing to an imbalance in bone remodeling. This study identifies neddylation, a post-translational modification, as a key regulator of this imbalance, affecting both osteoclast and osteoblast differentiation. Researchers found elevated NEDD8 conjugation in bone tissues from patients with PMOP and then evaluated MLN4924, a neddylation inhibitor, in a preclinical model. In ovariectomized mice, MLN4924 preserved bone mass by inhibiting osteoclast activity and promoting osteoblast function, highlighting its dual regulatory role. The study reveals that neddylation stabilizes NFATc1, promoting osteoclastogenesis, while destabilizing Runx2, suppressing osteoblast maturation. These findings suggest that targeting neddylation could offer a novel therapeutic approach for PMOP, addressing both excessive bone resorption and impaired bone formation.

This summary was initially drafted using artificial intelligence, then revised and fact-checked by the author.

## Introduction

Postmenopausal osteoporosis (PMOP) is a major public health challenge, affecting approximately one-third of women following menopause and leading to debilitating fractures in older adults^1,2^. This systemic skeletal disorder is characterized by progressive bone loss and microarchitectural deterioration owing to excessive osteoclast-mediated bone resorption over osteoblast-driven bone formation^3^. Despite this dual dysregulation, many current therapies target primarily one arm of bone remodeling imbalance. Nitrogen-containing bisphosphonates induce osteoclast apoptosis^4^, whereas denosumab blocks receptor activator of nuclear factor κB ligand (RANKL)-RANK signaling to prevent osteoclast differentiation^5^. By contrast, bone anabolic agents such as teriparatide, a parathyroid hormone analog, and abaloparatide, a parathyroid hormone-related protein analog, act as agonists of the parathyroid hormone 1 receptor and promote bone formation^6^. Romosozumab, an anti-sclerostin antibody, exerts both anabolic and antiresorptive effects by relieving sclerostin-mediated inhibition of Wnt/β-catenin signaling^7^. However, long-term antiresorptive therapy is associated with serious complications, including atypical femoral fractures and osteonecrosis of the jaw^8^, whereas anabolic and dual-effect agents remain limited by treatment duration or sequential-treatment requirements, safety concerns, and cost^9,10^. Collectively, these challenges underscore the need for new strategies that address both arms of this dysregulation.

Addressing this challenge requires a deeper understanding of the molecular mechanisms underlying bone remodeling imbalance. Although PMOP research has traditionally focussed on estrogen deficiency as the primary driver, emerging evidence highlights the importance of non-hormonal pathways^11^. Estrogen deficiency promotes T cell-derived RANKL production and induces nuclear factor of activated T cells, cytoplasmic 1 (NFATc1) in osteoclasts, thereby enhancing bone resorption^12^. It also suppresses osteoblastic markers including alkaline phosphatase (ALP) and runt-related transcription factor 2 (Runx2), leading to net bone loss^13^. Beyond these hormonal mechanisms, age-related epigenetic regulation and osteoimmune signaling also modulate bone remodeling independently of hormonal status^14–16^. Among these non-hormonal mechanisms, post-translational modifications (PTMs) have emerged as critical regulators. Although various PTMs such as acetylation, ubiquitination, and SUMOylation have been shown to regulate master transcription factors including NFATc1 and Runx2^17,18^, no single PTM pathway has been shown to simultaneously target both osteoclast and osteoblast differentiation to restore bone remodeling balance. Identifying such a PTM pathway represents an important step toward developing therapies that restore skeletal homeostasis.

Among PTMs, neddylation has emerged as a key regulator of cell fate decisions. Neddylation is a PTM whereby neural precursor cell expressed developmentally downregulated protein 8 (NEDD8) is covalently conjugated to target substrates through an E1–E2–E3 enzymatic cascade^19^. This process controls protein stability, localization, and activity to regulate lineage-specific gene expression programs during cellular differentiation^20^. Neddylation has been shown to regulate diverse cell fate decisions, including adipogenesis through PPARγ stabilization^21^, T cell activation via Shc-ERK signaling^22^, and myoblast differentiation through regulation of class Ⅱa/Ⅲ HDACs^23^. Beyond its role in normal differentiation, neddylation is also implicated in disease pathogenesis: dysregulated neddylation contributes to cancer progression, immune disorders, and metabolic diseases^24–26^. Despite its established functions in both cellular differentiation and disease, its role in bone cell differentiation and its therapeutic potential in PMOP remain unexplored.

In this study, we demonstrate that NEDD8 conjugation is elevated in bone tissue from patients with PMOP and identified distinct neddylation pathways that reciprocally regulate osteoclast and osteoblast differentiation. c-Cbl-mediated neddylation of NFATc1 promotes osteoclastogenesis, whereas Rbx1-mediated neddylation of Runx2 suppresses osteoblast maturation. To test this therapeutically, we used MLN4924, an inhibitor of the NEDD8-activating enzyme E1 subunit 1 (NAE1). Treatment with MLN4924 preserved trabecular microarchitecture and bone mass in ovariectomized (OVX) mice. These findings identify neddylation as a key regulator of bone remodeling imbalance in PMOP and highlight its potential as a therapeutic target.

## Materials and methods

### Human bone tissue acquisition

Human femoral heads were obtained during total hip arthroplasty or bipolar hemiarthroplasty at Seoul National University Hospital. The study was approved by the Institutional Review Board (IRB No. 2408-085-1560) and conducted in accordance with the Declaration of Helsinki. Informed consent was obtained from all participants or their legal representatives. A total of 42 female patients (aged 19–90 years) were enrolled. To ensure group clarity between PMOP and non-osteoporotic controls, bone mineral density (BMD) was assessed at the proximal femur and lumbar spine (L1–L4) using dual-energy X-ray absorptiometry, and patients were classified based on *T*-scores of the proximal femur: osteoporosis (*T* < –2.5), osteopenia (–2.5 ≤ *T* ≤ –1.0), and non-osteoporotic (*T* > –1.0). Twelve patients were diagnosed with osteoporosis and six were classified as normal controls. The detailed characteristics of the study subjects are summarized in Supplementary Table 1. Each femoral head was sectioned ~2 cm below the articular surface. For histological analysis, a portion of the tissue was fixed in 4% paraformaldehyde, decalcified in 10% EDTA for 3 months, and embedded in paraffin following institutional tissue handling protocols. Hematoxylin and eosin (H&E) staining was performed according to standard protocols. The remaining tissue was snap-frozen in liquid nitrogen and lysed in protein extraction buffer (iNtRON Biotechnology, Seongnam, Korea). Lysates were incubated on ice for 30 min and centrifuged at 12,000×*g* for 15 min; the lipid layer was removed, and the supernatant was collected for protein analysis.

### Animals and experimental models

C57BL/6 mice (KOATECH, Seoul, Korea) were carefully housed in a specific pathogen-free facility, maintaining a 12-h light/12-h dark cycle and constant room temperature and humidity. Eight-week-old female C57BL/6 mice were placed under isoflurane-induced anesthesia and underwent ovariectomy or SHAM surgery after a 1-week adaptation period. To analyze protein expression in bone tissue, a subset of mice was euthanized 4 weeks post-surgery, and femurs were collected. To evaluate the therapeutic effect of MLN4924 on osteoporosis, mice received intraperitoneal injections of MLN4924 (20 mg/kg) starting 1 week after OVX. The compound was administered once every 3 days for a total of 4 weeks. Following the 4-week treatment period, blood samples were collected from mice under respiratory anesthesia. Afterwards, the mice were euthanized, and each leg was used for micro-computed tomography (micro-CT), frozen sections and paraffin sections, respectively. H&E staining was performed according to standard protocols.

### Isolation of bone marrow-derived macrophages and osteoclast differentiation

Bone marrow-derived macrophages (BMMs) were obtained by flushing bone marrow cells from mouse femurs and tibiae using complete α-minimal essential media (MEM) (α-MEM supplemented with 10% fetal bovine serum (FBS) (Gibco) and 1% penicillin/streptomycin (Hyclone)). After straining through a 100 μm strainer, cells were centrifuged at 1500 × *g* for 5 min. Following the removal of red blood cell with RBC lysis buffer (Sigma-Aldrich, St Louis, MO, USA), the isolated cells were seeded in a cell culture dish. After obtaining the supernatant the next day, 30 ng/ml of recombinant mouse macrophage colony-stimulating factor (M-CSF) was added to the petri dish, and the cells were differentiated into macrophages for 3 days. After detaching the cells from the dish using a scraper, osteoclast differentiation was initiated with osteoclast differentiation media (Oc-DM). This involved adding 100 ng/ml of RANKL and 20 ng/ml of M-CSF to complete α-MEM. The Oc-DM was changed daily for 4 days. RAW 264.7 mouse macrophage cells were obtained from American Type Culture Collection (ATCC, Manassas, VA, USA) and cultured in α-MEM (α-MEM supplemented with 10% FBS (Gibco) and 1% penicillin/streptomycin). Osteoclast differentiation was initiated with α-MEM (α-MEM supplemented with 10% FBS (Gibco) and 1% penicillin/streptomycin) containing 100 ng/ml of RANKL.

### Primary osteoblast isolation and osteoblast differentiation

Primary osteoblasts (pOBs) were isolated from calvaria of newborn mice. Calvariae were dissected from skulls, and any attached soft tissue was removed, then minced into small pieces. Using α-MEM media supplemented with 3% penicillin/streptomycin, and containing 0.1% collagenase (Fujifilm Wako Pure Chemical Corporation, Japan) and 0.2% Dispase (Roche, Mannheim, Germany), calvaria were thoroughly digested through five repeated cycles. After straining through a 100 μm strainer, the cells were seeded into a cell culture dish. For pOB differentiation, cells were cultured in osteoblast differentiation medium (Ob-DM) (complete α-MEM supplemented with 10% FBS (Gibco) and 1% penicillin/streptomycin) containing 150 ng/ml of recombinant mouse BMP2, 50 μg/ml of ascorbic acid, and 10 mM β-glycerophosphate. C2C12 mouse mesenchymal precursor cells were obtained from ATCC and cultured in Dulbecco's modified Eagle's medium (DMEM) (DMEM supplemented with 10% FBS (Opti-Gold) and 1% penicillin/streptomycin). Osteoblast differentiation was initiated with DMEM (DMEM supplemented with 5% FBS (Gibco) and 1% penicillin/streptomycin) containing 100 ng/ml of recombinant mouse BMP2, 50 μg/ml of ascorbic acid, 10 mM β-glycerophosphate, and 10 μM dexamethasone.

### Tartrate-resistant acid phosphatase (TRAP) staining

TRAP staining was performed on paraffin sections or cultured cells. Sections and cells were treated with fixation solution (7.5 mM citrate solution, 3.7% formaldehyde solution in acetone) for 15 min. After washing, cells were exposed to the mixture of staining solution in acid phosphatase, leukocyte (TRAP) kit (Sigma-Aldrich, Merck, Darmstadt, Germany). Staining results were observed under a microscope. For in vitro analysis, mature osteoclasts were defined as TRAP-positive multinucleated cells (>3 nuclei/cell) and the number of osteoclasts was counted. For in vivo analysis, histomorphometric analysis was performed in the trabecular bone region of the distal femoral metaphysis, and osteoclast surface/bone surface (Oc.S/BS) and osteoclast number/bone perimeter (N.Oc/B.Pm) were quantified from TRAP-stained paraffin sections using ImageJ software.

### ALP staining

For ALP staining, cells and frozen sections were fixed with fixation solution (7.5 mM citrate solution and 3.7% formaldehyde solution in acetone) for 5 min. Fixed cells and frozen sections were rinsed with distilled water and incubated at 37 °C for 10 min in the 5-bromo-4-chloro-3-indolyl phosphate and nitro blue tetrazolium solution (Sigma-Aldrich). For in vivo analysis, histomorphometric assessment was performed in the trabecular region of the distal femoral metaphysis. ALP-positive osteoblasts lining the trabecular bone surface were identified and counted, and osteoblast number per bone perimeter (N.Ob/B.Pm) was calculated. Osteoblast surface per bone surface (Ob.S/BS) was determined as the ratio of ALP-positive osteoblast perimeter to total bone perimeter using ImageJ software.

### Alizarin Red S (ARS) staining

For ARS staining, samples were fixed with 3.7% formaldehyde for 15 min. Samples were rinsed with distilled water and stained with 2% ARS (pH 4.2) at 37 °C for 10 min. After capturing images, the precipitated minerals from the stained samples were extracted to quantify ARS staining of cells, and their concentration was assessed using absorbance measurements. The staining samples were incubated in 10% acetic acid at room temperature for 30 min with shaking. The cells were then transferred to a microcentrifuge tube and boiled at 85 °C for 10 min. After centrifugation at 20,000×*g* for 15 min, the acid was neutralized with 10% ammonium hydroxide, and the absorbance was measured at 405 nm using a microplate reader.

### ALP activity assay

Following the incubation of the cell lysate with *p*-nitrophenyl phosphate (pNPP, Sigma-Aldrich) at 37 °C for 1 h, the ALP activity was measured by quantifying the absorbance at 405 nm. To quantify the enzyme activity in the samples, a *p*-nitrophenol standard curve was established using different concentrations of pNPP (ranging from 0 nmol to 20 nmol) and 10 units of calf intestine ALP (Fermentas). The absorbance values obtained from the standard curve were then utilized to determine the ALP activity levels in the experimental samples.

### Masson trichrome staining

Paraffin-embedded tissue sections were subjected to deparaffinization and rehydration before being treated with Bouin’s solution at 56 °C for 60 min. Subsequently, the sections were washed with distilled water and stained using Weigert’s iron hematoxylin for 10 min. The samples underwent rinsing with distilled water and exposure to Biebrich scarlet-acid fuchsin solution for 8 min, followed by phosphotungstic-phosphomolybdic acid solution for 10 min. Samples were then incubated in aniline blue solution and 1% acetic acid. After this process, the tissue sections underwent dehydration before mounting.

### Immunofluorescence and phalloidin analysis

For immunofluorescence staining of cultured cells, cells were fixed with 4% paraformaldehyde for 10 min and permeabilized with phosphate-buffered saline (PBS) containing 0.3% Triton X-100. After blocking with PBS containing 3% bovine serum albumin for 1 h at room temperature, cells were incubated with primary antibodies overnight at 4 °C. After three washes with PBS, fluorophore-conjugated secondary antibodies were applied for 1 h at room temperature. For phalloidin staining, cells were fixed, permeabilized, and blocked as described earlier, followed by incubation with fluorophore-conjugated phalloidin (Invitrogen, Carlsbad, CA, USA) for 40 min at room temperature. Following antibody or phalloidin labeling, nuclei were counterstained with 4′,6-diamidino-2-phenylindole (DAPI), and coverslips were mounted using FluorSave™ mounting medium (Merck Millipore, Billerica, MA, USA). Images were acquired using a confocal microscope (FV3000; Olympus, Tokyo, Japan).

### Immunofluorescence analysis for human bone tissue

For human tissue sections, paraffin-embedded femoral-head samples were sectioned, deparaffinized, and rehydrated. Antigen retrieval was performed by boiling the sections in 10 mM sodium citrate buffer (pH 6.0) in a microwave for 20 min. After blocking with 10% normal goat serum for 1 h at room temperature, the sections were incubated overnight at 4 °C with primary antibodies against NEDD8 (Cell Signaling Technology, 2745S, 1:200), NFATc1 (Abcam, ab2796, 1:200), and Runx2 (Abcam, ab236639, 1:200). Alexa Fluor 647-conjugated antibodies against osteocalcin (R&D System, Minneapolis, MN, USA) and cathepsin K (Novus, Centennial, CO, USA) were also applied and incubated overnight at 4 °C. Where appropriate, secondary antibodies conjugated with fluorophores were applied for 1 h at room temperature. DAPI was used for nuclear counterstaining, and slides were mounted with FluorSave™ mounting medium. Confocal images were acquired using the FV3000 system (Olympus).

### Bone histology and immunohistochemistry

For frozen sections, tissues were fixed overnight in 4% paraformaldehyde after micro-CT imaging and decalcified for 3 days in 10% ethylenediaminetetraacetic acid (EDTA) (pH 7.2–7.5). Decalcified samples were incubated overnight at 4 °C in 15% and 30% sucrose successively, embedded in OCT compound (Leica), and sectioned at 8 μm. Sections were stained with ALP. For paraffin sections, femurs were fixed and decalcified, embedded in paraffin, and sectioned at 4 μm for H&E staining. For immunostaining, deparaffinized sections underwent antigen retrieval by microwave heating in 10 mM sodium citrate buffer (pH 6.0) for 20 min. Sections were blocked with 10% normal goat serum for 1 h, incubated overnight at 4 °C with antibodies, followed by biotinylated secondary antibody incubation for 1 h. Visualization was performed using ABC kit (VECTOR, Burlingame, CA, USA) and DAB (DAKO, Denmark), followed by hematoxylin counterstaining. *H*-scores were calculated as weighted averages of staining intensity (1, 2, or 3 for weak, moderate, or strong, respectively) and positive cell proportion. Three trabecular regions per slide were scored and averaged. Quantification used ImageJ software (NIH). Pearson’s and Spearman’s correlation analyses were performed using R v.4.4.2.

### Micro-CT analysis

Micro-CT of mice femur was performed using a Quantum GX2 μCT system (PerkinElmer, Waltham, MA, USA). Dissected mouse femurs were fixed in 4% paraformaldehyde for 24 h. Micro-CT scanning was performed using an X-ray tube potential of 80 kV, an X-ray intensity of 100 μA, and an exposure time of 14 min with a field of view of 10 mm (voxel size, 20 μm). The 3D images were visualized with a 3D viewer consisting of software within the Quantum GX2. The Analyze 12.0 software (AnalyzeDirect, USA) was used to measure bone parameters.

### Statistical analysis

All experiments were repeated on at least three independent occasions and analyzed using Microsoft Excel (Microsoft Corp., Redmond, WA, USA), GraphPad Prism 8 (GraphPad Inc., La Jolla, CA, USA), or R v.4.4.2 (https://www.r-project.org/) software. Results are expressed as means ± s.d. or median (IQR), as appropriate. Two-tailed unpaired *t* tests and one-way analysis of variance were used for normally distributed data, and the Mann–Whitney *U*-test for non-normal data. *P*-values less than 0.05 were considered statistically significant.

Additional methods are described in the Supplementary information.

## Results

### Neddylation is elevated in PMOP

**Fig. 1:**
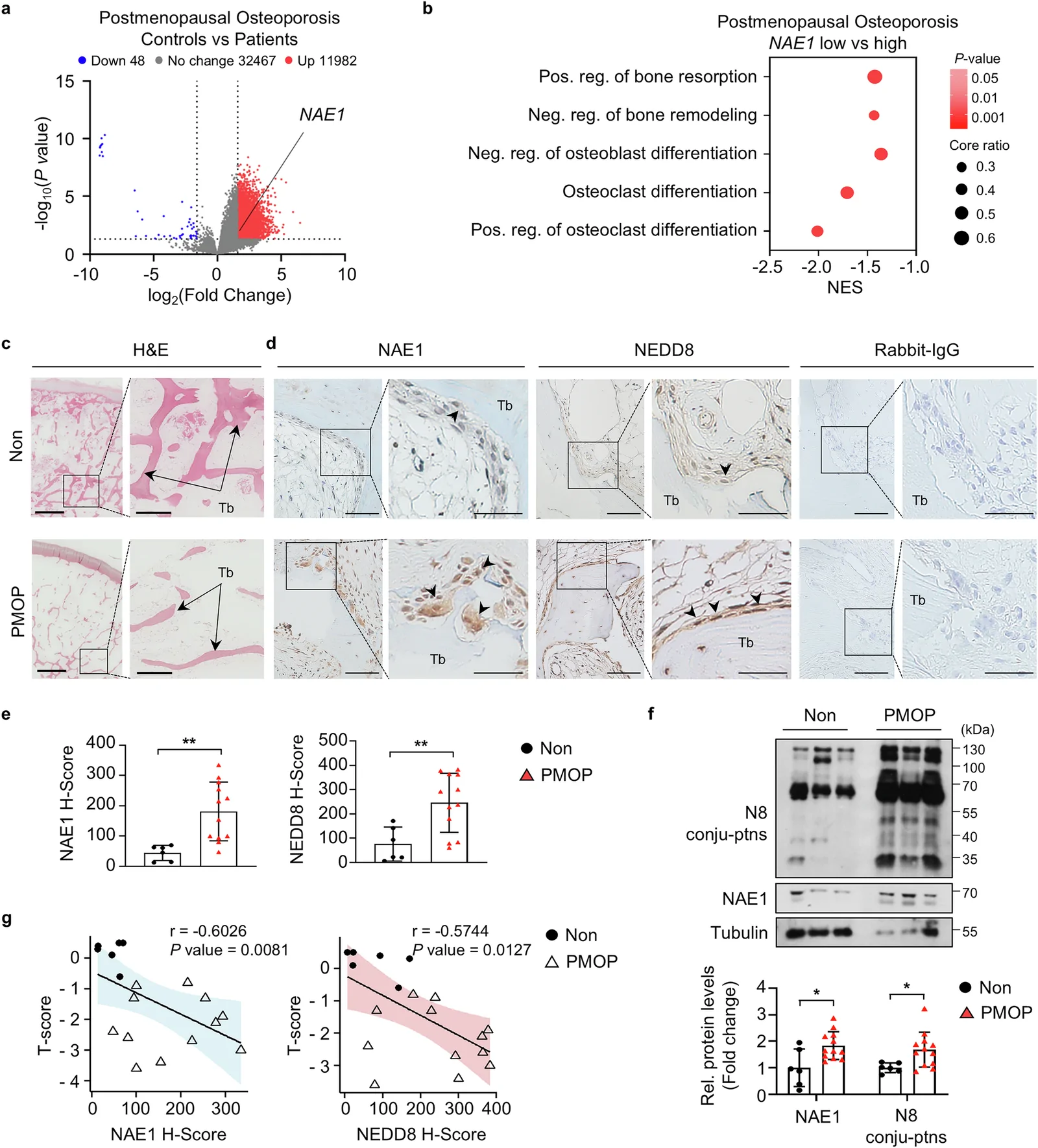
Neddylation is elevated in bone from patients with PMOP. **a**, The volcano plot displays the differentially expressed genes between non-osteoporotic controls (*n* = 3) and patients with postmenopausal osteoporosis (PMOP) (*n* = 12) from dataset GSE230665. Dashed lines denote thresholds (log2 Fold Change > 1.6 or < –1.6; –log10 *P* > 1.3); *NAE1* is highlighted in the plot. **b**, Bubble plot of gene set enrichment analysis (GSEA) comparing *NAE1* expression quartiles; comparisons are shown for *NAE1* high (highest quartile) versus *NAE1* low (lowest quartile). The *x*-axis shows normalized enrichment score (NES); dot size indicates core ratio (core genes/total genes); color encodes nominal *P*-value. Gene set names and detailed enrichment statistics (NES, nominal *P*) are provided in Supplementary Fig. 1. **c**, Representative hematoxylin and eosin (H&E) images of femoral heads from non-osteoporotic controls (Non, *n* = 6) and patients with PMOP (*n* = 12). Left, low magnification; right, high magnification. Scale bars, 4 mm (left) and 1 mm (right). **d**, Representative immunohistochemistry (IHC) for NAE1, NEDD8, and IgG control in femoral heads (Non, *n* = 6; PMOP, *n* = 12). Arrowheads indicate positive cells. Left, low magnification; right, high magnification. Scale bars, 100 µm (left) and 50 µm (right). **e**, Quantification of IHC staining by *H*-score (Non, *n* = 6; PMOP, *n* = 12). Statistical significance was determined by a two-sided Mann–Whitney *U*-test. **f**, Representative immunoblots of NAE1 and NEDD8-conjugated proteins (N8 conju-ptns), in femoral head lysates from non-osteoporotic controls and patients with PMOP. Densitometry was performed in ImageJ, normalized to tubulin (Tub), and expressed relative to the Non group (Non, *n* = 6; PMOP, *n* = 12). Statistical significance was determined by a two-sided Mann–Whitney *U-*test. N8 conju-ptns: NEDD8-conjugated proteins. **g**, Correlations between *T*-scores and *H*-scores for NAE1 or NEDD8 as in parts **d** and **e**. Spearman’s correlation coefficient (*r*) and *P*-values were determined by linear regression; shaded area, 95% confidence interval. The data are presented as the mean ± s.d.; *P < 0.05; **P < 0.01. Neg, negative; Pos, positive; reg, regulation; Tb, trabecular bone.

To assess neddylation in PMOP, we first examined NAE1 expression as a proxy for pathway activity. NAE1, in conjunction with UBA3, forms the NEDD8-activating enzyme (E1), which initiates neddylation by activating NEDD8 in an ATP-dependent manner^27^. We analyzed the GSE230665 transcriptome dataset comprising non-osteoporotic controls (*n* = 3) and patients with PMOP (*n* = 12). Volcano plot analysis revealed a significant increase in *NAE1* mRNA levels in patient samples compared with controls (Fig. 1a). To further investigate the relationship between neddylation and bone remodeling, we performed gene set enrichment analysis (GSEA). Quartile-based stratification indicated that high *NAE1* expression was associated with enrichment of gene sets promoting bone resorption and osteoclast differentiation, as well as gene sets involved in the negative regulation of bone remodeling and osteoblast differentiation (Fig. 1b and Supplementary Fig. 1). To assess whether neddylation is upregulated in bone tissue from women with PMOP, we analyzed femoral-head samples from women with PMOP and from non-osteoporotic female controls (Non) classified according to dual-energy X-ray absorptiometry-derived *T*-scores (Supplementary Fig. 2). H&E staining confirmed the trabecular bone loss characteristic of osteoporosis (Fig. 1c). Immunohistochemistry showed that NAE1 and NEDD8 expression levels were significantly elevated in the trabecular regions of osteoporotic bone compared with non-osteoporotic bone (Fig. 1d, e). Immunoblot analysis of bone tissue revealed increased levels of NEDD8-conjugated proteins and NAE1 (Fig. 1f). Additionally, quantitative analysis of immunohistochemistry staining showed that NAE1 and NEDD8 *H*-scores negatively correlated with *T*-scores (Fig. 1g). We then validated these findings in an animal model by inducing osteoporosis in mice via OVX and analyzing the distal femur 1 month after surgery. H&E staining revealed a reduction in trabecular bone beneath the growth plate. Additionally, immunohistochemistry confirmed increased NAE1 and NEDD8 expression levels in the trabecular region of OVX mice (Supplementary Fig. 3a–c). Immunoblot analysis similarly showed elevated levels of NEDD8-conjugated proteins and NAE1 in OVX mice, consistent with human data (Supplementary Fig. 3d). Collectively, these findings indicate increased NAE1 and NEDD8 expression in PMOP, suggesting that neddylation is elevated.

### Neddylation reciprocally regulates the differentiation of osteoclasts and osteoblasts

**Fig. 2:**
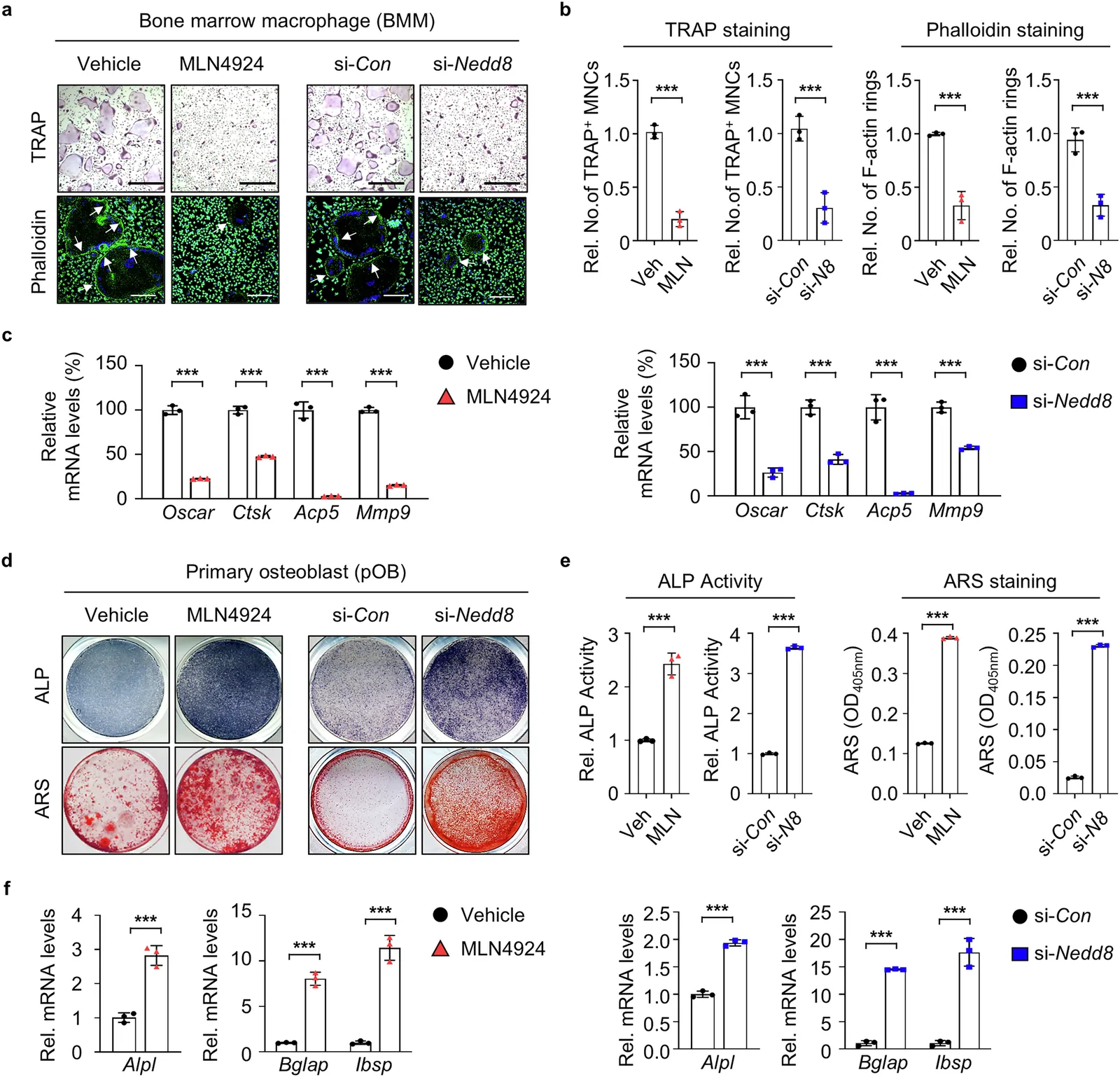
Neddylation reciprocally regulates osteoclast and osteoblast differentiation. **a**, Osteoclasts (OCs) derived from BMMs were treated with either vehicle, MLN4924 (50 nM), control siRNA, or si-*Nedd8* and cultured in OC differentiation medium for 4 days. Tartrate-resistant acid phosphatase (TRAP) staining (top) shows TRAP^+^ multinucleated cells (MNCs; ≥3 nuclei). Phalloidin staining (bottom) visualizes F-actin rings. Scale bars, 500 µm (TRAP) and 200 µm (phalloidin). **b**, Quantification of TRAP^+^ MNCs and F-actin rings in part **a**. **c**, Total RNAs were isolated from BMMs treated as in part **a**. The expression of OC differentiation marker genes was quantified by RT-qPCR. **d**, Primary osteoblasts (pOBs) were cultured in osteoblast differentiation medium and treated with vehicle, MLN4924 (50 nM), si-*Con*, or si-*Nedd8*. Representative alkaline phosphatase (ALP) staining (day 3) and Alizarin Red S (ARS) staining (day 5). **e**, Quantification of ALP activity and mineral deposition (ARS absorbance at 405 nm) in pOBs treated as in part **d**. **f**, Total RNAs were isolated from pOBs treated as in part **d**. The expression of OB marker genes was quantified using RT-qPCR (*Alpl* on day 3 and *Bglap* and *Ibsp* on day 5). The data are presented as mean ± s.d. (*n* = 3); n.s., not significant; *P < 0.05; **P < 0.01; ***P < 0.001. Statistical significance was determined by two-tailed unpaired *t* tests.

Bone remodeling depends on the balanced activity of osteoclasts and osteoblasts. Accordingly, we investigated whether neddylation affects their differentiation. We derived BMMs from mouse femora in the presence of M-CSF and isolated pOBs from mouse calvariae. To induce differentiation, we cultured BMMs in Oc-DM and pOBs in Ob-DM. Neddylation inhibition via MLN4924, also known as pevonedistat, treatment, or si-*Nedd8* transfection significantly impaired the differentiation and maturation of osteoclasts in BMMs and RAW 264.7 cells (Fig. 2a, b and Supplementary Fig. 4a). Consistent with this finding, blocking neddylation reduced the mRNA levels of osteoclast marker genes, including *Oscar*, cathepsin K (*Ctsk*), TRAP (*Acp5*), and *Mmp9* (Fig. 2c and Supplementary Fig. 4b). By contrast, in osteoblasts, these treatments significantly promoted differentiation in pOBs and C2C12 cells, as evidenced by increased ALP activity and mineralization (Fig. 2d, e and Supplementary Fig. 4c). Consistently, we observed increased mRNA levels of osteogenic markers, including *Alpl*, osteocalcin (*Bglap*), and *Ibsp* (Fig. 2f and Supplementary Fig. 4d). Together, these results demonstrate that neddylation blockade reciprocally regulates bone cell differentiation by inhibiting osteoclastogenesis while promoting osteoblastogenesis.

### NFATc1 destabilization by neddylation blockade impairs osteoclast differentiation

**Fig. 3:**
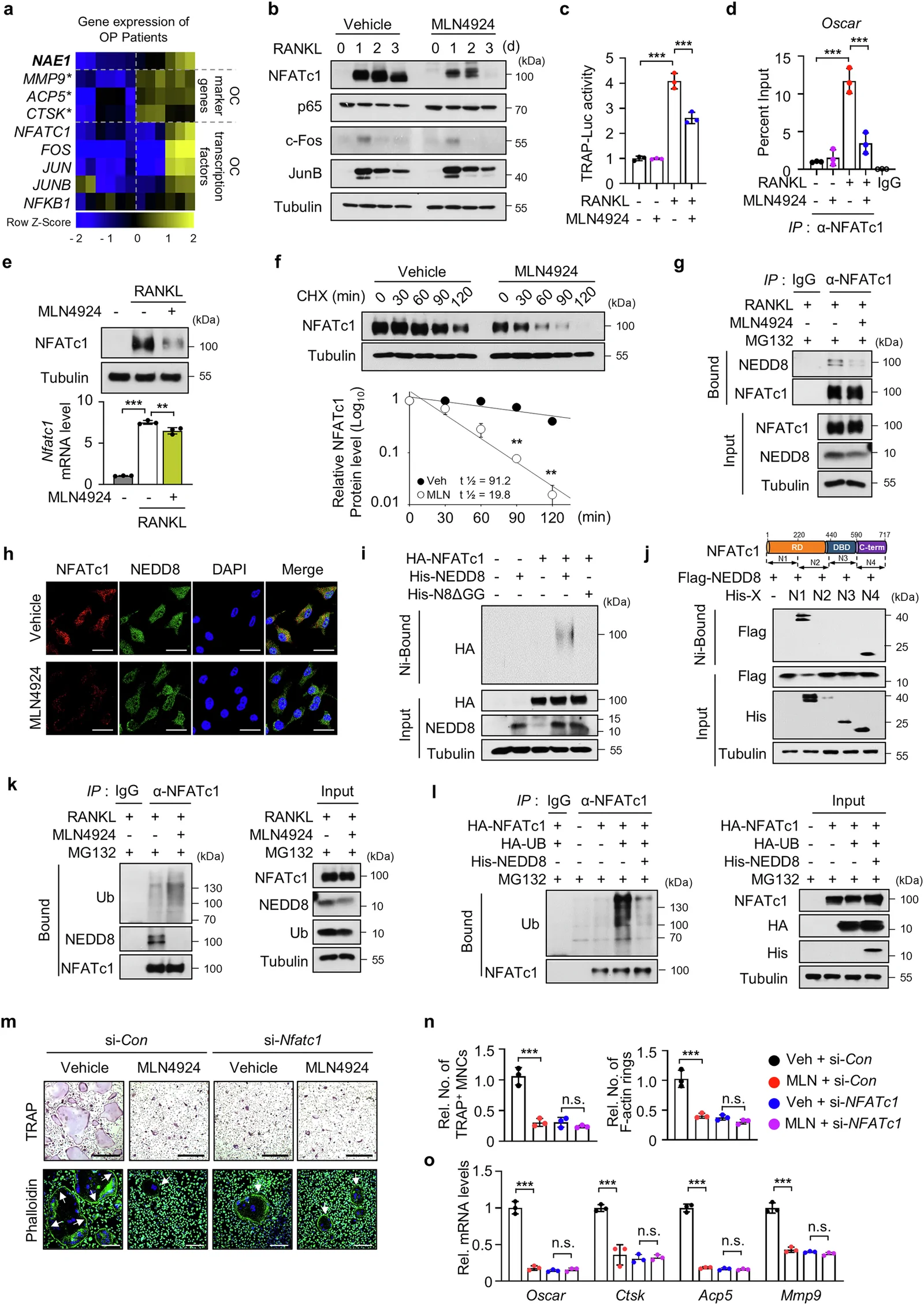
Neddylation enhances the stability of NFATc1 protein during osteoclast differentiation. **a**, Heat map of GSE230665 showing osteoclast (OC) markers and transcription factors expression stratified by *NAE1* (median split). Row *Z*-scores: low (blue) to high (yellow). **b**, Lysates from bone marrow-derived macrophages (BMMs) cultured in osteoclast differentiation medium (Oc-DM) with vehicle or MLN4924 (50 nM) were immunoblotted for NFATc1, p65, c-Fos, and JunB. **c**, Tartrate-resistant acid phosphatase (TRAP)-luciferase activity in RAW 264.7 cells treated with RANKL (100 ng/ml) and MLN4924 for 1 day. **d**, ChIP-qPCR shows NFATc1 occupancy at the *Oscar* promoter in RAW 264.7 cells treated as in part **c**. **e**, Immunoblot and RT-qPCR of NFATc1 were performed on BMMs cultured in Oc-DM ± MLN4924 for 2 days. **f**, BMMs cultured in Oc-DM ± MLN4924 for 2 days were treated with cycloheximide (CHX; 100 µM) for the indicated times. NFATc1 protein level was quantified by immunoblot densitometry, and half-life (*t*_½_) was calculated by linear regression of log_10_-transformed values. **g**, Lysates from BMMs treated as in part **e** with MG132 (10 µM) for the final 3 h were immunoprecipitated with anti-NFATc1 and immunoblotted for NEDD8 and NFATc1. **h**, Immunofluorescence shows NFATc1 (red) and NEDD8 (green) in BMMs (Oc-DM ± MLN, 2 days). Scale bar, 20 µm. **i**, Lysates from HEK293T cells co-transfected with HA-NFATc1 and either His-NEDD8 or His-NEDD8ΔGG were subjected to Ni^2+^ pull-down. **j**, NFATc1 domain mapping (N1–N4) with Flag-NEDD8 was performed using Ni^2+^ pull-down. Immunoprecipitation showing NFATc1 interaction with ubiquitin (Ub) and NEDD8 from BMMs (part **k**, treated as in part **g**) and HEK293T cells (part **l**, with MG132, 6 h). **m**, TRAP (top) and phalloidin (bottom) staining were performed in BMMs transfected with si-*Con* or si-*Nfatc1* and cultured with Oc-DM ± MLN for 4 days (arrows, F-actin rings; scale bars, 500 µm (TRAP) and 200 µm (phalloidin)). **n**, TRAP^+^ multinucleated cells (MNCs) and F-actin rings were quantified. **o**, RT-qPCR shows OC marker genes in BMMs treated as in part **m**. The data are presented as mean ± s.d. (*n* = 3); n.s., not significant; **P < 0.01; ***P < 0.001. Statistical significance was determined by two-tailed unpaired *t* test (parts **a** and **f**) or one-way analysis of variance followed by Bonferroni’s post hoc test (parts **c**–**e**, **n**, and **o**). C-term, C-terminal domain; DAPI, 4′,6-diamidino-2-phenylindole; DBD, DNA-binding domain; OP, osteoporosis; RD, regulatory domain.

Neddylation regulates diverse cellular processes by modifying various substrates, including transcription factors that control cell differentiation^28^. Given that MLN4924 treatment reduced both osteoclast differentiation and marker gene expression (Fig. 2a–c), we hypothesized that neddylation might regulate key transcription factors controlling osteoclastogenesis. To test this, we re-analyzed the GSE230665 dataset, stratifying samples based on the median *NAE1* mRNA level. We found that osteoclast marker genes were significantly elevated in the *NAE1*-high group, whereas transcription factor genes showed no consistent change (Fig. 3a). At the protein level, immunoblotting during osteoclast differentiation showed that among major transcription factors, only the NFATc1 protein level was markedly reduced by MLN4924, suggesting post-translational regulation of NFATc1 (Fig. 3b). Consistent with reduced NFATc1 protein, a TRAP-luciferase reporter assay revealed reduced NFATc1 transcriptional activity, and ChIP-qPCR showed diminished NFATc1 binding to the *Oscar* promoter, a canonical NFATc1 target locus during osteoclastogenesis^29^ (Fig. 3c, d and Supplementary Fig. 5). In BMMs, MLN4924 treatment decreased NFATc1 protein level, whereas *Nfatc1* mRNA showed only a slight reduction (Fig. 3e). Cycloheximide (CHX) chase assays demonstrated accelerated turnover of NFATc1 in the presence of MLN4924, suggesting that neddylation maintains NFATc1 stability (Fig. 3f). We next examined whether NFATc1 is directly neddylated. Immunoprecipitation revealed that endogenous NEDD8 was conjugated to NFATc1 during osteoclast differentiation (Fig. 3g). Immunofluorescence further showed colocalization of NFATc1 and NEDD8 during osteoclastogenesis (Fig. 3h). Ni^2+^-NTA pull-down assays confirmed covalent conjugation of HA-NFATc1 by His-NEDD8, but not by the conjugation-defective His-NEDD8ΔGG mutant (Fig. 3i). Domain mapping localized NEDD8 attachment to the N-terminal regulatory domain and C-terminal domains of NFATc1 (Fig. 3j). As NFATc1 undergoes ubiquitin-mediated proteasomal degradation^30–32^, we investigated whether neddylation affects this process. Immunoprecipitation assays showed that neddylation blockade by MLN4924 increased NFATc1 ubiquitination in BMMs treated with Oc-DM, whereas overexpression of His-NEDD8 reduced NFATc1 ubiquitination in HEK293T cells (Fig. 3k, l). *Nfatc1* knockdown abrogated the suppressive effects of MLN4924 on osteoclastogenesis, such that MLN4924 no longer reduced TRAP⁺ multinucleated cell formation and F-actin ring assembly (Fig. 3m, n). Similarly, *Nfatc1* knockdown prevented the MLN4924-induced downregulation of osteoclast marker genes (*Oscar*, *Ctsk*, *Acp5*, and *Mmp9*) (Fig. 3o). Together, these findings show that neddylation directly stabilizes NFATc1, thereby preventing its ubiquitin-dependent proteasomal degradation and promoting osteoclast differentiation.

### c-Cbl acts as a neddylation E3 ligase for NFATc1

**Fig. 4:**
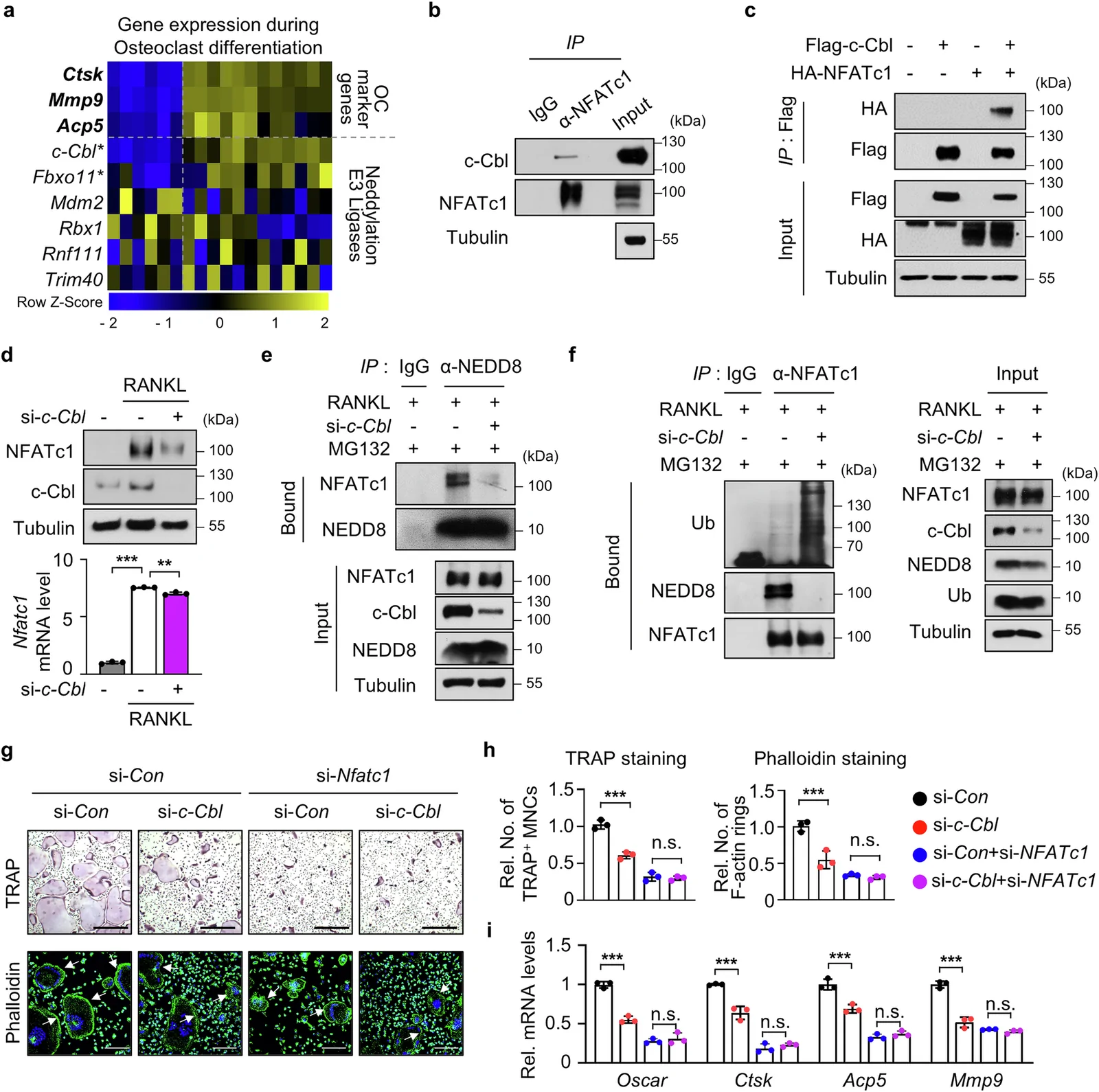
c-Cbl neddylates NFATc1 as an E3 ligase. **a**, Heat map of GSE176265 shows neddylation-related E3 ligases and osteoclast (OC) marker genes. Samples were clustered by OC marker expression (high versus low) using *k*-means clustering. Row *Z*-scores: low (blue) to high (yellow). **b**, Lysates from bone marrow-derived macrophages (BMMs) cultured in osteoclast differentiation medium (Oc-DM) for 2 days were immunoprecipitated with anti-NFATc1 and immunoblotted for c-Cbl. **c**, Lysates from HEK293T cells co-transfected with Flag-c-Cbl and HA-NFATc1 were immunoprecipitated with anti-Flag and immunoblotted with anti-HA and anti-Flag. **d**, BMMs transfected with si-*Con* or si-*c-Cbl* and cultured in Oc-DM for 2 days were analyzed by immunoblot for NFATc1 and c-Cbl (top), and *Nfatc1* mRNA was quantified by real-time-quantitative PCR (bottom). **e**, Lysates from BMMs transfected with si-*Con* or si-*c-Cbl*, cultured in Oc-DM for 2 days, and treated with MG132 (10 µM) for the final 3 h were immunoprecipitated with anti-NEDD8 or IgG and immunoblotted for NFATc1. **f**, Lysates from BMMs treated as in part **e** were immunoprecipitated with anti-NFATc1 or IgG and immunoblotted for ubiquitin (Ub) and NEDD8. **g**, Tartrate-resistant acid phosphatase (TRAP) (top) and phalloidin (bottom) staining were performed in BMMs transfected with si-*Con*, si-*c-Cbl*, si-*Nfatc1*, or combination, and cultured in Oc-DM for 4 days (arrows, F-actin rings; scale bars, 500 µm (TRAP) and 200 µm (phalloidin)). **h**, TRAP^+^ multinucleated cells (MNCs) and F-actin rings were quantified. **i**, RT-qPCR shows OC marker genes in BMMs treated as in part **g**. The data are presented as mean ± s.d. (*n* = 3); n.s., not significant; **P < 0.01; ***P < 0.001. Statistical significance was determined by two-tailed unpaired *t* test (part **a**) or one-way analysis of variance followed by Bonferroni’s post hoc test (parts **d**, **h**, and **i**).

Identifying the E3 ligase responsible for neddylation of NFATc1 is crucial for mechanistic understanding, because E3 ligases confer substrate specificity and represent potential therapeutic targets^33^. We therefore sought to identify candidate neddylation E3 ligases for NFATc1 by analyzing the GSE176265 dataset. Using *k*-means clustering to divide the samples into groups with high and low osteoclast marker expression, we found that *c-Cbl* and *Fbxo11* were enriched in the high-expression group (Fig. 4a). Because c-Cbl has been implicated in osteoclast regulation^34,35^, we focused on c-Cbl. Co-immunoprecipitation experiments conducted with BMMs revealed an interaction between endogenous NFATc1 and c-Cbl (Fig. 4b). This interaction was also observed in HEK293T cells that expressed both Flag-c-Cbl and HA-NFATc1 (Fig. 4c). Knockdown of *c-Cbl* decreased NFATc1 protein level with minimal effect on *Nfatc1* mRNA (Fig. 4d). *c-Cbl* depletion reduced the association between NFATc1 and NEDD8 and increased NFATc1 ubiquitination (Fig. 4e, f), indicating that *c-Cbl*-mediated neddylation protects NFATc1 from ubiquitin-dependent proteasomal degradation. We next investigated whether *c-Cbl* knockdown mimics the effects of MLN4924. Silencing *c-Cbl* led to a reduction in the formation of TRAP⁺ multinucleated cells and the assembly of F-actin rings, whereas concurrent knockdown of *Nfatc1* abolished these effects (Fig. 4g, h). Similarly, *c-Cbl* knockdown did not affect the expression of osteoclast marker genes (*Oscar*, *Ctsk*, *Acp5*, and *Mmp9*) in NFATc1-depleted cells (Fig. 4i). These findings identify *c-Cbl* as a neddylation E3 ligase that stabilizes NFATc1 and promotes osteoclast differentiation.

### Runx2 stabilization by neddylation blockade promotes osteoblast differentiation

**Fig. 5:**
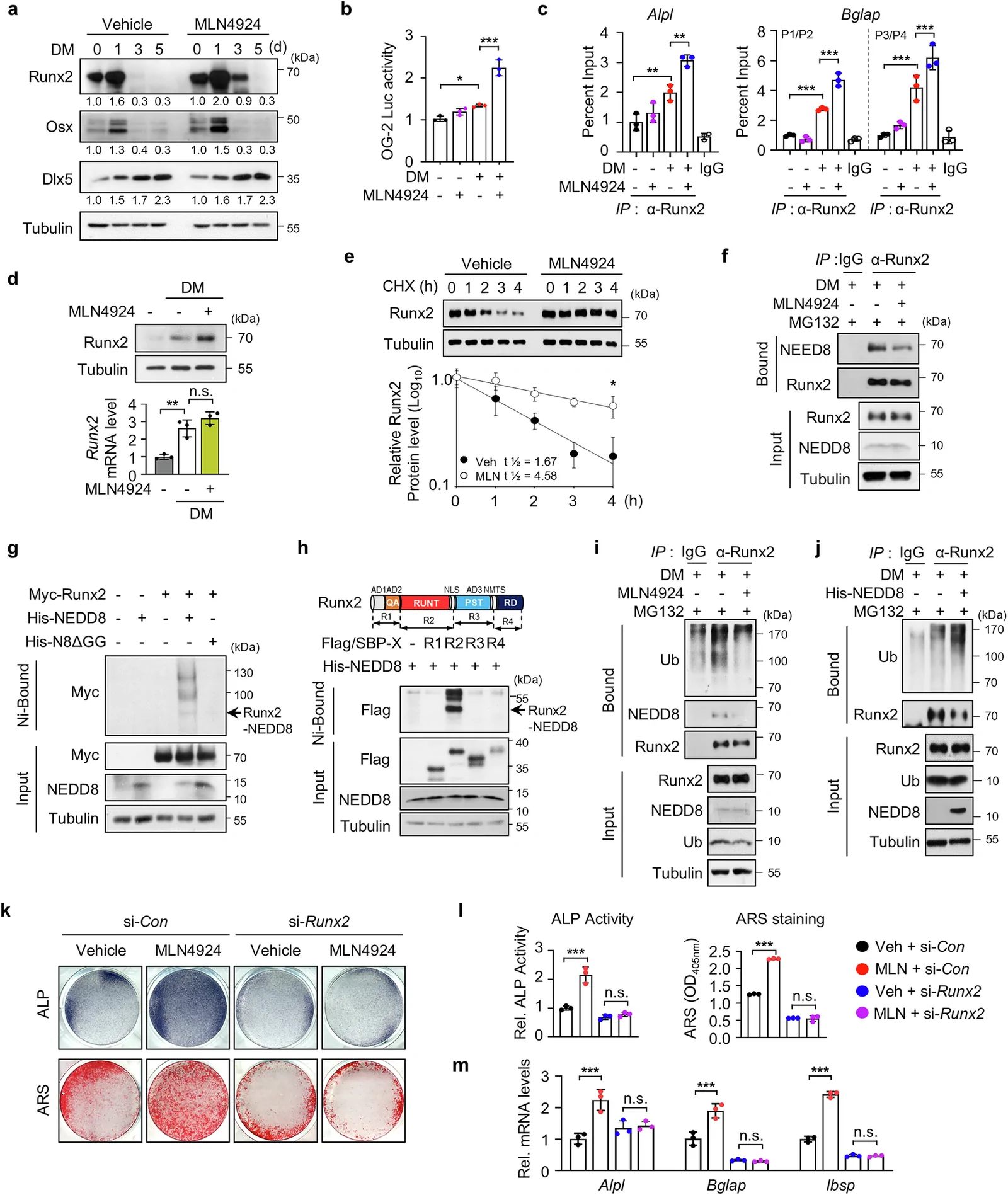
Neddylation destabilizes Runx2 protein during osteoblast differentiation. **a**, Lysates from primary osteoblasts (pOBs) cultured in osteoblast differentiation medium (Ob-DM) with vehicle or MLN4924 (50 nM) were immunoblotted for Runx2, Osx, and Dlx5. Numbers below the blots indicate relative band intensities normalized to Tubulin and expressed relative to day 0, which was set to 1. **b**, OG2-luciferase activity in C2C12 cells treated with Ob-DM ± MLN4924 for 2 days. **c**, ChIP-qPCR shows Runx2 occupancy at the *Alpl* enhancer and the *Bglap* promoters in C2C12 cells treated as in part **b**. **d**, Immunoblot and RT-qPCR of Runx2 were performed on pOBs cultured in Ob-DM ± MLN4924 for 1 day. **e**, C2C12 cells cultured in Ob-DM ± MLN4924 for 1 day were treated with cycloheximide (CHX; 100 µM) for the indicated times. Runx2 protein level was quantified by immunoblot densitometry, and half-life (*t*_½_) was calculated by linear regression of log₁₀-transformed values. **f**, Lysates from pOBs treated as in part **d** with MG132 (10 µM) for the final 6 h were immunoprecipitated with anti-Runx2 and immunoblotted for NEDD8 and Runx2. **g**, Lysates from HEK293T cells co-transfected with Myc-Runx2 and either His-NEDD8 or His-NEDD8ΔGG were subjected to Ni²⁺ pull-down assay. **h**, Runx2 domain mapping (R1–R4) with His-NEDD8 was performed using Ni²⁺ pull-down assay. Immunoprecipitation showing Runx2 interaction with ubiquitin (Ub) and NEDD8 from pOBs (part **i**) and C2C12 cells (part **j**) treated as in (part **f**). **k**, Alkaline phosphatase (ALP) (top) and Alizarin Red S (ARS) (bottom) staining were performed in pOBs transfected with si-*Con* or si-*Runx2* and cultured with Ob-DM ± MLN (ALP, 3 days; ARS, 5 days). **l**, Quantification of ALP activity (left) and ARS absorbance (right) is shown. **m**, RT-qPCR shows OB marker genes (*Alpl* day 3; *Bglap*, *Ibsp* day 5) in pOBs treated as in part **k**. The data are presented as the mean ± s.d. (*n* = 3); n.s., not significant; *P < 0.05; **P < 0.01; ***P < 0.001. Statistical significance was determined by two-tailed unpaired *t* test (part **e**) or one-way analysis of variance followed by Bonferroni’s post hoc test (parts **b**–**d**, **l**, and **m**). PST, proline/serine/threonine-rich domain; QA, glutamine/alanine-rich domain; RD, repression domain; Runt, Runt homology domain.

Having established that neddylation inhibition promotes osteoblast differentiation (Fig. 2d–f), we next investigated the underlying mechanism. To identify the neddylation substrate, we analyzed GSE181459 by stratifying samples based on the median *Nae1* mRNA level. Osteoblast markers (*Alpl*, *Bglap*, and *Ibsp*) were higher in the *Nae1*-low group, whereas key transcription factors (*Runx2*, *Osx*, and *Dlx5*) remained unchanged (Supplementary Fig. 6a). During osteoblast differentiation, MLN4924 treatment selectively increased Runx2 protein level, whereas Osx and Dlx5 remained unchanged (Fig. 5a and Supplementary Fig. 6b). Runx2 transcriptional activity increased in OG2-luciferase reporter assays, and ChIP-qPCR revealed enhanced Runx2 occupancy at *Alpl* and *Bglap* regulatory elements (Fig. 5b, c and Supplementary Fig. 5). MLN4924 elevated Runx2 protein level without altering *Runx2* mRNA level (Fig. 5d). CHX chase assay revealed that MLN4924 markedly extended the half-life of Runx2 (Fig. 5e), in contrast to NFATc1 (Fig. 3f). To determine whether Runx2 itself undergoes neddylation, immunoprecipitation detected endogenous neddylated Runx2 in differentiating osteoblasts (Fig. 5f). Ni^2+^-NTA pull-down assays using His-NEDD8 confirmed covalent conjugation to Runx2, and domain mapping localized this modification to the Runt domain (Fig. 5g, h). Because Runx2 is also subject to ubiquitin-dependent degradation^36,37^, we investigated whether neddylation modulates this process. Indeed, MLN4924 treatment reduced endogenous Runx2 ubiquitination, whereas ectopic expression of NEDD8 increased Runx2 ubiquitination (Fig. 5i, j). We next tested whether the MLN4924-induced osteoblast differentiation requires Runx2. *Runx2* knockdown abolished the MLN4924-induced increase in ALP activity and mineralized-nodule formation (Fig. 5k, l), and the expression of osteoblast markers (*Alpl*, *Bglap*, and *Ibsp*) was not increased by MLN4924 in Runx2-depleted cells (Fig. 5m). Together, these results demonstrate that neddylation blockade stabilizes Runx2 to promote osteoblast differentiation, revealing a mechanism reciprocal to NFATc1 destabilization in osteoclasts.

### Rbx1 functions as a neddylation E3 ligase for Runx2

**Fig. 6:**
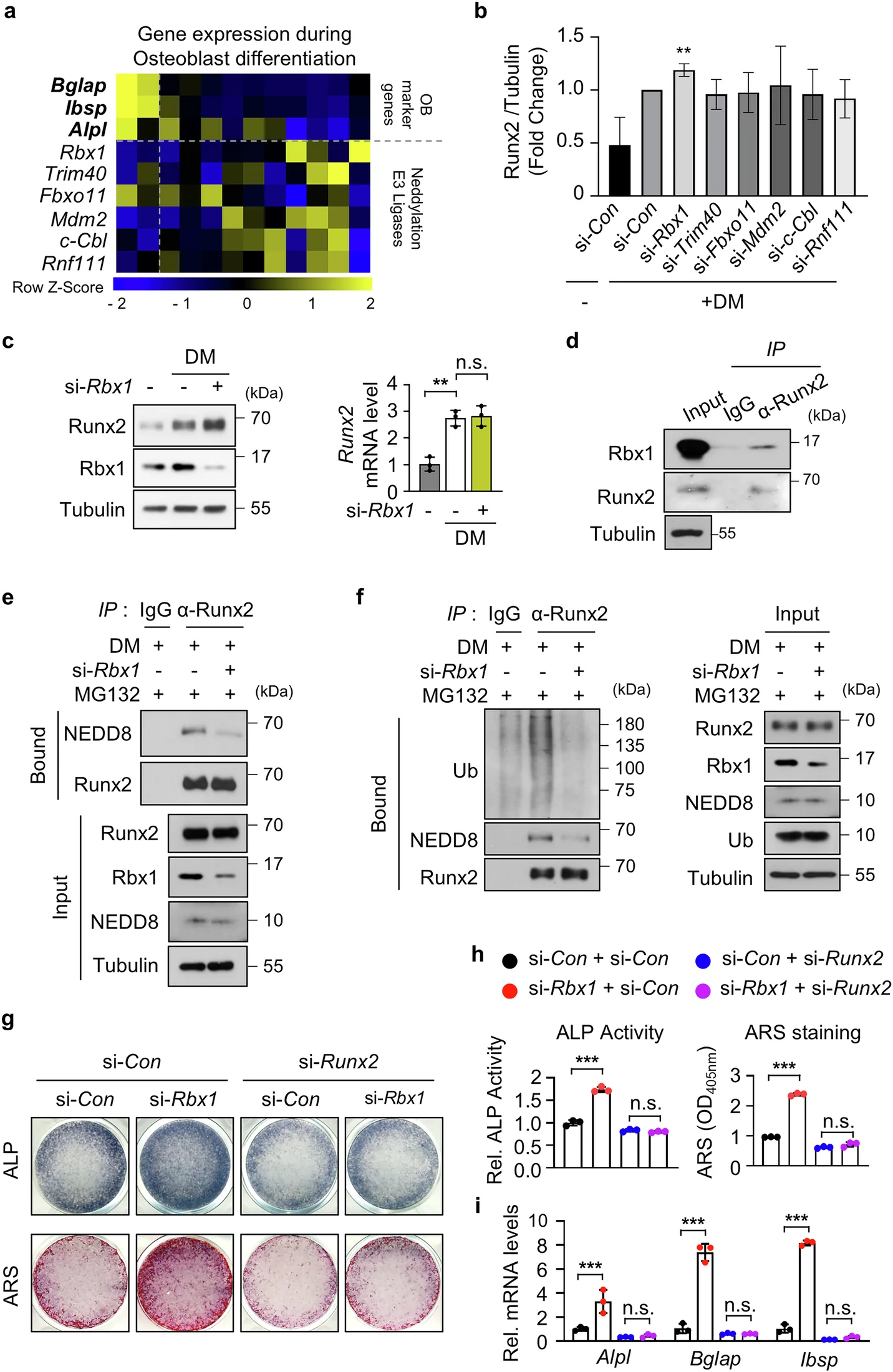
Rbx1 neddylates Runx2 as an E3 ligase. **a**, Heat map of GSE181459 shows neddylation-related E3 ligases and osteoblast (OB) marker genes. Samples were clustered by OB marker expression (high versus low) using *k*-means clustering. Row *Z*-scores: low (blue) to high (yellow). **b**, Runx2 was quantified from immunoblots (from Supplementary Fig. 7b) of C2C12 cells transfected with si-*Con* or si-*E3 ligases*. Values were normalized to tubulin and expressed as fold change relative to si-*Con* + Ob-DM group. **c**, Primary osteoblasts (pOBs) transfected with si-*Con* or si-*Rbx1* and cultured in osteoblast differentiation medium (Ob-DM) for 1 day were analyzed by immunoblot for Runx2 and Rbx1 (left), and *Runx2* mRNA was quantified by RT-qPCR (right). **d**, Lysates from pOBs cultured in Ob-DM for 1 day were immunoprecipitated with anti-Runx2 and immunoblotted for Rbx1. **e**, Lysates from pOBs transfected with si-*Con* or si-*Rbx1*, cultured in Ob-DM for 1 day, and treated with MG132 (10 µM) for the final 6 h were immunoprecipitated with anti-Runx2 or IgG and immunoblotted for NEDD8. **f**, Lysates from pOBs treated as in part **e** were immunoprecipitated with anti-Runx2 or IgG and immunoblotted for ubiquitin (Ub) and NEDD8. **g**, Alkaline phosphatase (ALP) (top) and Alizarin Red S (ARS) (bottom) staining were performed in pOBs transfected with si-*Con*, si-*Rbx1*, si-*Runx2*, or the combination, and cultured in Ob-DM for 3 days (ALP) or 5 days (ARS). **h**, Quantification of ALP activity (left) and ARS absorbance (right) is shown. **i**, RT-qPCR shows OB marker genes (*Alpl* day 3; *Bglap*, *Ibsp* day 5) in pOBs treated as in part **g**. Data are presented as mean ± s.d. (*n* = 3); n.s., not significant; **P < 0.01; ***P < 0.001. Statistical significance was determined by two-tailed unpaired *t* test (part **b**) or one-way analysis of variance followed by Bonferroni’s post hoc test (parts **c**, **h**, and **i**).

To identify candidate E3 ligases for Runx2 neddylation, we first analyzed the GSE181459 dataset and, using *k*-means clustering, classified samples into groups with high and low osteoblast marker expression. No candidate neddylation E3 ligase showed expression patterns that correlated with osteoblast markers (Fig. 6a), prompting us to perform a functional RNA interference screen. Among the candidates, *Rbx1* silencing showed the largest increase in Runx2 protein during osteoblast differentiation, whereas knockdown of other E3 ligases had little effect (Fig. 6b and Supplementary Fig. 7a, b). *Rbx1* knockdown elevated Runx2 protein level while leaving *Runx2* mRNA unchanged (Fig. 6c). Co-immunoprecipitation revealed an interaction between Rbx1 and Runx2 in pOBs (Fig. 6d). *Rbx1* depletion reduced the Runx2–NEDD8 interaction and decreased Runx2 ubiquitination (Fig. 6e, f). These findings suggest that Rbx1-mediated neddylation of Runx2 promotes its ubiquitin-dependent proteasomal degradation. Functionally, *Rbx1* knockdown enhanced osteoblast differentiation, as shown by increased ALP activity and mineralized-nodule formation, whereas simultaneous *Runx2* silencing abolished these effects (Fig. 6g, h). The induction of osteoblast marker genes (*Alpl*, *Bglap*, and *Ibsp*) by *Rbx1* depletion was also lost upon *Runx2* silencing (Fig. 6i). Collectively, these findings identify Rbx1 as a neddylation E3 ligase for Runx2 that targets Runx2 for degradation and suppresses osteoblast differentiation.

### MLN4924 treatment preserves bone mass in OVX mice

**Fig. 7:**
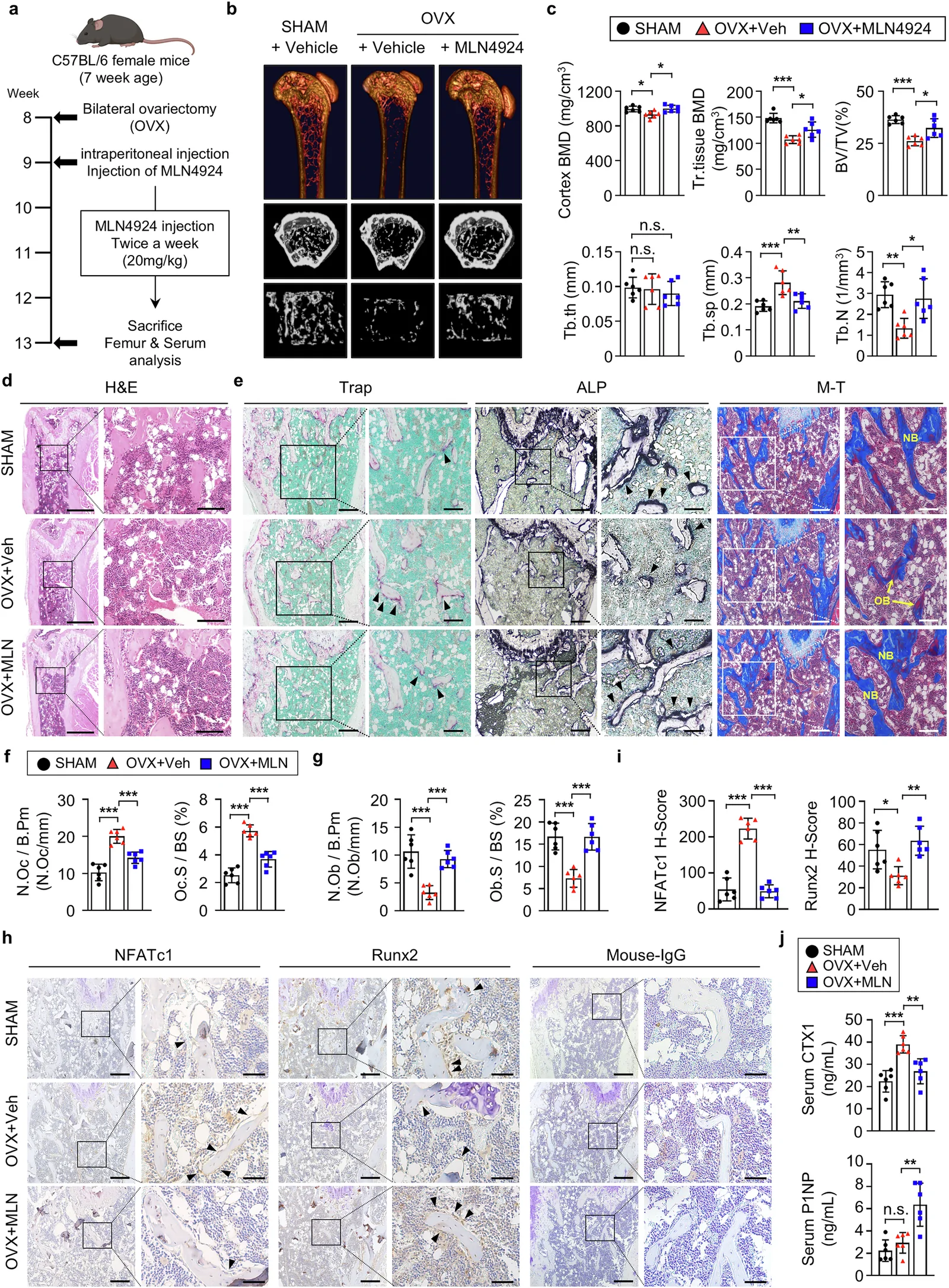
MLN4924 ameliorates OVX-induced bone loss and promotes bone formation in vivo. **a**, Schematic diagram of the experimental model. Eight-week-old female C57BL/6 mice underwent either a sham operation (SHAM) or ovariectomy (OVX), followed by intraperitoneal MLN4924 (MLN, 20 mg/kg) or vehicle twice weekly for 4 weeks (*n* = 6 independent animals in each group). **b**, Representative micro-CT images of distal femora from SHAM, OVX, and OVX + MLN groups (top, longitudinal view; middle and bottom, cross-sectional and 3D trabecular reconstruction of the distal metaphyseal region). **c**, Quantitative micro-CT analysis of distal femora: bone mineral density (BMD), bone volume/total volume (BV/TV), trabecular thickness (Tb.Th), trabecular separation (Tb.Sp), and trabecular number (Tb.N). **d**, Representative hematoxylin and eosin (H&E) images of distal femoral sections from each group. Left, low magnification; right, high magnification. Scale bars, 500 µm (left) and 100 µm (right). **e**, Tartrate-resistant acid phosphatase (TRAP), alkaline phosphatase (ALP), and Masson trichrome (M-T) staining of femoral sections. Left, low magnification; right, high magnification. Arrowheads indicate positive signals. Scale bars, 200 µm (left) and 100 µm (right). **f**, Quantification of osteoclast number per bone perimeter (N.Oc/B.Pm, N.Oc/mm) (left) and osteoclast surface per bone surface (Oc.S/BS, %) (right) in the TRAP-stained femoral sections shown in part **e**. **g**, Quantification of osteoblast number per bone perimeter (N.Ob/B.Pm, N.Ob/mm) (left) and osteoblast surface per bone surface (Ob.S/BS, %) (right) in the ALP-stained femoral sections shown in part **e**. **h**, Representative immunohistochemistry for NFATc1, Runx2, and IgG control in femoral sections. Arrowheads indicate positive cells. Left, low magnification; right, high magnification. Scale bars, 200 µm (left) and 100 µm (right). **i**, Quantification of immunohistochemical staining by *H*-score. **j**, Serum C-terminal telopeptide of type I collagen (CTX-1) and procollagen type I N-terminal propeptide (P1NP) measured by enzyme-linked immunosorbent assay. The data are presented as mean ± s.d. (*n* = 6 independent animals in each group). n.s., not significant; *P < 0.05; **P < 0.01; ***P < 0.001. Statistical significance was determined by one-way analysis of variance followed by Bonferroni’s post hoc test. NB, new bone; OB, old bone.

To evaluate the therapeutic potential of neddylation inhibition in vivo, we utilized an OVX mouse model of PMOP. Eight-week-old C57BL/6 female mice underwent sham surgery or OVX and subsequently received intraperitoneal injections of vehicle or MLN4924 (20 mg/kg, twice weekly) for 4 weeks (Fig. 7a). Micro-CT imaging of the distal femur revealed that OVX mice exhibited a marked reduction in bone mass compared with SHAM controls, whereas MLN4924 treatment (OVX + MLN) preserved trabecular microarchitecture (Fig. 7b and Supplementary Fig. 8a–c). Quantitative analysis showed higher cortical and trabecular BMD, BV/TV, and Tb.N and lower Tb.Sp in the OVX + MLN group compared with vehicle-treated OVX controls (OVX + Veh), with Tb.Th unchanged (Fig. 7c and Supplementary Fig. 8d). H&E staining confirmed these microarchitectural improvements, demonstrating dense trabecular structure in the OVX + MLN group (Fig. 7d). Histological staining further showed that MLN4924 reduced TRAP^+^ area and increased ALP-positive area, with Masson trichrome staining confirming enhanced new-bone deposition in treated mice (Fig. 7e). Consistent with these staining results, N.Oc/B.Pm and Oc.S/BS were increased in OVX mice and reduced by MLN4924, whereas N.Ob/B.Pm and Ob.S/BS were decreased in OVX mice and restored by MLN4924 (Fig. 7f, g). Immunohistochemical analysis showed decreased NFATc1 expression and increased Runx2 expression in MLN4924-treated mice (Fig. 7h, i). Notably, NAE1 and NEDD8 expression levels remained elevated in both OVX + Veh and OVX + MLN groups compared with SHAM controls, with no significant reduction by MLN4924 treatment (Supplementary Fig. 9a, b). Serum biomarker analysis supported these histological findings, showing that MLN4924 treatment significantly reduced serum CTX-1 level, a marker of bone resorption, and elevated P1NP level, a marker of bone formation (Fig. 7j). Collectively, these results demonstrate that pharmacological inhibition of neddylation preserves bone mass in OVX mice by reciprocally regulating osteoclast and osteoblast activity.

### Neddylation drives reciprocal NFATc1 and Runx2 dysregulation in osteoclast and osteoblast lineages in PMOP

**Fig. 8:**
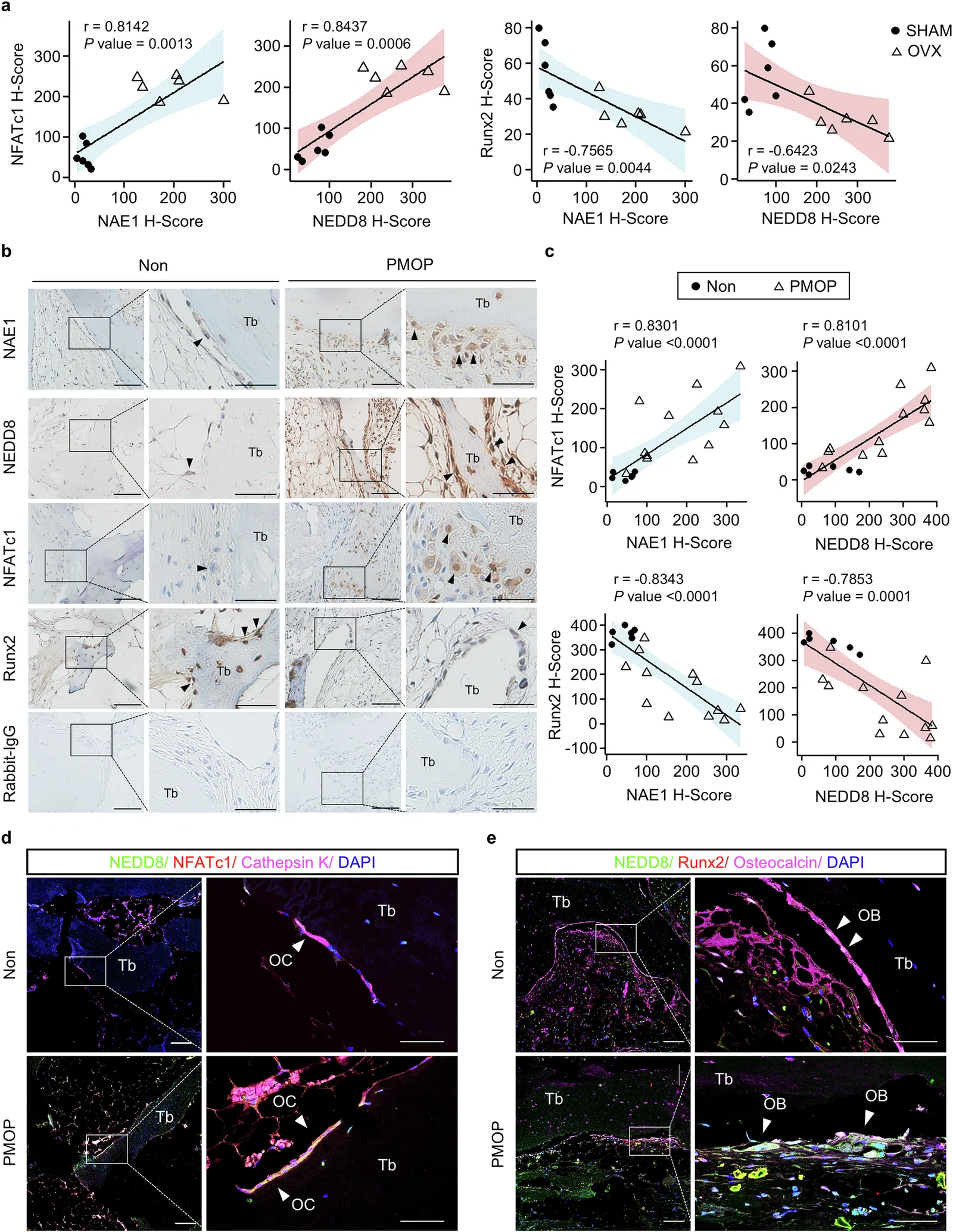
Neddylation reciprocally dysregulates NFATc1 and Runx2 in PMOP bone. **a**, Correlations between immunohistochemical (IHC) *H*-scores for NAE1 or NEDD8 and those for NFATc1 (left) or Runx2 (right) in sham operation (SHAM) (*n* = 6) and ovariectomy (OVX)+Veh (*n* = 6) groups from the OVX mouse experiment shown in Fig. 7. Pearson’s correlation coefficient (*r*) and *P*-values were determined by linear regression; shaded area, 95% confidence interval. **b**, Representative IHC for NAE1, NEDD8, NFATc1, Runx2, and IgG control in femoral heads from non-osteoporotic controls (Non, *n* = 6) and patients with postmenopausal osteoporosis (*n* = 12). Arrowheads indicate positive cells. Left, low magnification; right, high magnification. Scale bars, 100 µm (left) and 50 µm (right). **c**, Correlations between *H*-scores of NAE1 or NEDD8 and NFATc1 (top) or Runx2 (bottom) across PMOP and Non samples. Spearman’s correlation coefficient (*r*) and *P*-values were determined by linear regression; shaded area, 95% confidence interval. **d**, Immunofluorescence for NEDD8 (green), NFATc1 (red), and cathepsin K (magenta) with 4′,6-diamidino-2-phenylindole (DAPI) (blue) in femoral head sections from each group. Arrowheads indicate cathepsin K-positive osteoclasts (OCs) adjacent to trabecular (Tb). Left, low magnification; right, high magnification. Scale bars, 150 µm (left) and 50 µm (right). **e**, Immunofluorescence for NEDD8 (green), Runx2 (red), and osteocalcin (magenta) with DAPI (blue). Arrowheads indicate osteocalcin-positive osteoblasts (OBs) adjacent to Tb. Left, low magnification; right, high magnification. Scale bars, 150 µm (left) and 50 µm (right).

To validate these mechanisms and to assess their translational relevance, we first examined whether immunohistochemical *H*-scores for NFATc1 and Runx2, reflecting protein expression levels, were correlated with those for NAE1 and NEDD8 in our OVX mouse model. In SHAM and OVX + Veh groups, the *H*-scores of NAE1 and NEDD8 were positively associated with NFATc1 but negatively associated with Runx2 (Fig. 8a). Notably, these correlations were attenuated in MLN4924-treated group (Supplementary Fig. 10), suggesting that the therapeutic benefit of neddylation blockade operates through the regulation of NFATc1 and Runx2 levels. We next analyzed NFATc1 and Runx2 expression in femoral-head samples from patients with PMOP and non-osteoporotic (Non) controls. Immunohistochemical analysis revealed elevated expression of NAE1 and NEDD8 in PMOP samples compared with Non, along with increased NFATc1 and decreased Runx2 expression (Fig. 8b and Supplementary Fig. 11). Correlation analysis demonstrated that NAE1 and NEDD8 levels positively correlated with NFATc1 but negatively correlated with Runx2 in human bone tissue (Fig. 8c), consistent with disease-associated neddylation dysregulation. To further characterize these changes in osteoclasts and osteoblasts, we performed immunofluorescence co-staining of NEDD8 with NFATc1/cathepsin K (osteoclast markers) and with Runx2/osteocalcin (osteoblast markers). In PMOP samples, NEDD8 and NFATc1 strongly colocalized in cathepsin K-positive osteoclasts (Fig. 8d). Conversely, in osteoblasts, increased NEDD8 coincided with reduced Runx2 and osteocalcin expression compared with Non (Fig. 8e). Together, these findings support an association between increased neddylation pathway activity and reciprocal dysregulation of NFATc1 and Runx2 in osteoclast-lineage and osteoblast-lineage cells in OVX mice and human PMOP bone. The attenuation of these changes by MLN4924 in OVX mice highlights the translational potential of targeting neddylation in PMOP.

## Discussion

Current therapies for PMOP primarily target osteoclast activity but fail to restore osteoblast function and therefore do not address the underlying imbalance between excessive bone resorption and impaired bone formation^38^. This therapeutic gap stems from an incomplete understanding of the molecular mechanisms that coordinate this dual dysregulation. Although PTMs such as ubiquitination and SUMOylation modulate individual bone cell types^39,40^, no single pathway has been shown to reciprocally regulate the master transcription factors of both lineages. Here, we identify neddylation as a mechanism that reciprocally regulates both lineages. We found that NEDD8 conjugation is elevated in bone tissue from patients with PMOP and OVX mice, indicating heightened pathway activity in disease states (Fig. 1 and Supplementary Fig. 3). Mechanistically, neddylation stabilizes NFATc1 to promote osteoclastogenesis while destabilizing Runx2 to suppress osteoblast maturation, thereby coordinating both arms of the remodeling imbalance through a single PTM pathway (Figs. 3 and 5). Consistent with this dual role, pharmacological inhibition with MLN4924 simultaneously reduced bone resorption and preserved bone formation, maintaining trabecular architecture in OVX mice (Fig. 7). Together, these findings support a unifying role for neddylation in coupling osteoclast and osteoblast dysfunction in PMOP and highlight a therapeutically actionable target for rebalancing skeletal homeostasis.

NFATc1 is the master transcription factor that drives osteoclast differentiation^41^. NFATc1-null mice exhibit complete failure of osteoclastogenesis and severe osteopetrosis^42^. Given its essential role, multiple PTMs have been reported to regulate its stability and activity. For example, Cbl-b and c-Cbl ubiquitinate NFATc1 to promote its degradation in the later stages^30^. SUV39H1-mediated methylation facilitates its turnover, whereas acetylation by PCAF and p300 stabilizes NFATc1 and enhances its transcriptional activity^43,44^. We found that neddylation represents an additional regulatory layer in NFATc1 regulation. Specifically, c-Cbl functions as the neddylation E3 ligase that stabilizes NFATc1 during early osteoclastogenesis by counteracting its ubiquitin-dependent proteasomal degradation. This stabilization is mechanistically supported by the increased NFATc1 ubiquitination upon MLN4924 treatment and its reduction by ectopic NEDD8 expression (Fig. 3k, l), as well as by the effects of c-Cbl depletion on NFATc1 neddylation and ubiquitination (Fig. 4e, f). However, c-Cbl has been primarily characterized as a ubiquitin E3 ligase that promotes NFATc1 degradation in late-stage osteoclasts^30^, raising the question of how one E3 ligase mediates opposing effects. We propose that c-Cbl operates in a context-dependent manner. This dual functionality is consistent with findings in lung cancer cells, in which c-Cbl acts as both a neddylation and ubiquitination E3 ligase for EGFR: it recruits the NEDD8-conjugating enzyme UBE2M to neddylate EGFR while simultaneously functioning as a ubiquitin E3 ligase, with neddylation enhancing subsequent EGFR ubiquitination and degradation^45,46^. The ability of c-Cbl to apply both modifications to the same substrate suggests that similar context-dependent coordination may govern NFATc1 regulation during osteoclast differentiation.

Runx2 is the primary transcription factor of skeletal development, playing a crucial role in osteoblast differentiation^47^. Mice with heterozygous Runx2 mutation exhibit delayed ossification and reduced bone mass, suggesting that slight changes in Runx2 stability significantly impact skeletal development^48^. Multiple PTMs regulate Runx2 stability and activity: ubiquitin E3 ligases such as Smurf1 and WWP1 promote proteasomal degradation^49,50^, whereas interactions with CBFβ and demethylation enhance its stability and activity^51,52^. We found that neddylation represents an additional degradation-promoting modification of Runx2, with Rbx1 functioning as the responsible E3 ligase (Fig. 6). Rbx1 is best known as the RING component of cullin-RING E3 ubiquitin ligases, where it activates ubiquitin ligase complexes such as SCF or VHL through cullin neddylation^53^. Although Rbx1 has been extensively studied in cancer^54^, in which its elevated expression is linked to tumor progression, metastasis, and poor survival outcomes, its role in osteoblasts remains unclear. Our findings reveal Rbx1-mediated neddylation as a regulatory mechanism that destabilizes Runx2 to suppress osteoblast maturation (Fig. 6c, g). This destabilization is mechanistically attributable to facilitation of ubiquitin-dependent proteasomal degradation, as evidenced by increased Runx2 ubiquitination upon ectopic NEDD8 expression and its reduction by MLN4924 treatment (Fig. 5j, k). These findings identify Rbx1-dependent Runx2 neddylation as a potential therapeutic target for restoring bone formation in PMOP.

Neddylation was initially characterized in the activation of cullin-RING E3 ubiquitin ligases, but it is now recognized to modify a wide range of non-cullin proteins involved in diverse cellular processes^55^. The effects of neddylation are highly substrate-specific. In some cases, neddylation can promote ubiquitination-dependent degradation, as observed with c-Src and EGFR^45,56^. Conversely, neddylation stabilizes proteins by blocking ubiquitination, as demonstrated for PPARγ and SREBP1c^21,57^. This dual role highlights the flexibility of neddylation as a regulatory mechanism, with its effects depending on both the properties of the target protein and the cellular context. Our findings reveal that both regulatory modes operate within the bone microenvironment, but on different master transcription factors (Figs. 3f and 5e). This reciprocal regulation suggests that neddylation functions as a molecular switch that coordinately controls bone remodeling (Fig. 2). The opposing consequences of neddylation on NFATc1 and Runx2 provide a molecular basis for understanding how a single PTM can drive the coordinated dysregulation observed in PMOP.

The therapeutic potential of targeting neddylation is supported by our preclinical studies with MLN4924. MLN4924, also known as pevonedistat, is a selective NAE1 inhibitor that has advanced through phase I and II oncology studies across various schedules. Clinical studies have demonstrated on-target NAE1 inhibition in tumors of patients^58,59^. In adult patients with cancer, exposure levels at ~60 mg/m^2^ have been associated with increased liver enzymes (ALP and AST), making dose selection and careful monitoring crucial outside of oncology settings^60^. In our previous research, a 30 mg/kg dose of MLN4924 showed no significant toxicity in assessments that included body weight, behavioral activity, and liver histopathology^57^. Our study demonstrated that a 20 mg/kg dose maintained BMD and preserved trabecular architecture in OVX mice (Fig. 7a), with no significant body weight changes observed (data not shown). Nevertheless, because neddylation regulates diverse cellular processes in multiple cell types^28^, systemic inhibition of this pathway may carry potential adverse effects beyond bone^58^. Thus, although our findings support the therapeutic promise of neddylation inhibition in PMOP, they should be regarded as proof-of-concept, and further studies will be required to define long-term safety, optimal dosing, and strategies to improve tissue selectivity.

In conclusion, our study identifies neddylation as a central regulatory mechanism governing bone remodeling imbalance in PMOP. We demonstrate that c-Cbl-mediated neddylation stabilizes NFATc1 to amplify osteoclastogenesis, whereas Rbx1-mediated neddylation destabilizes Runx2 to suppress osteoblast maturation. Elevated NEDD8 and NAE1 expression in bone tissue from patients with PMOP, accompanied by increased NFATc1 and decreased Runx2, validates the clinical relevance of these pathways in human disease. Pharmacological inhibition of neddylation with MLN4924 simultaneously attenuates bone resorption and preserves bone formation, thereby preserving trabecular microarchitecture in OVX mice. These findings position neddylation as a therapeutically actionable target that addresses a fundamental limitation of current antiresorptive therapies by enabling dual control of skeletal homeostasis. Our work supports the clinical development of neddylation inhibitors as a promising therapeutic strategy for PMOP.

## Supplementary information

## Data Availability

All data needed to evaluate the conclusions in the paper are presented in the paper and the Supplementary information. The following publicly available datasets were analyzed in this study: GSE230665 (https://www.ncbi.nlm.nih.gov/geo/query/acc.cgi?acc=GSE230665), GSE176265 (https://www.ncbi.nlm.nih.gov/geo/query/acc.cgi?acc=GSE176265), and GSE181459 (https://www.ncbi.nlm.nih.gov/geo/query/acc.cgi?acc=GSE181459). Supplementary information is available online at the Experimental & Molecular Medicine website (http://www.nature.com/emm/).
