# Supplementary material for: Neddylation of NFATc1 and Runx2 regulates osteoclast–osteoblast balance and represents a dual-action therapeutic target for postmenopausal osteoporosis
Source: Exp Mol Med. 2026 Aug 3;58(8):2603–19. doi: 10.1038/s12276-026-01784-2 (PMC13538646; doi:10.1038/s12276-026-01784-2)
Supplement: Supplementary file 1 [file 12276_2026_1784_MOESM1_ESM.pdf]

Supplementary Information for

Neddylation of NFATc1 and Runx2 regulates osteoclast–osteoblast balance and represents a dual-action therapeutic target for postmenopausal osteoporosis

Jooseung Lee[1,3,*], Min Young Lee[1,3,*], Hong Seok Kim[4], Jong-Wan Park[1,2], Geon Ho Moon[1,3], Jun Bum Park[5], Hye-Jin Kim[1], Sung Joon Kim[1,2,3] and Yang-Sook Chun[1,2,3]

[1]Department of Biomedical Sciences, Seoul National University College of Medicine, Seoul, 03080, South Korea.

[2]Ischemic/Hypoxic Disease Institute, Seoul National University College of Medicine, Seoul, 03080, South Korea.

[3]Department of Physiology, Seoul National University College of Medicine, Seoul, 03080, South Korea.

[4]Department of Orthopedic Surgery, Seoul National University Hospital, Seoul National University College of Medicine, Seoul, 03080, South Korea.

[5]Department of Medicine, Massachusetts General Hospital, Harvard Medical School, Boston, MA, USA.

[*]These authors contributed equally to this work

Correspondence: Yang-Sook Chun, chunys@snu.ac.kr

Supplementary Methods

Plasmids, siRNA and transfection

PCR-amplified cDNAs of mouse NFATc1 were inserted into HA-tagged pcDNA3 (Clontech Laboratories, Mountain View, CA, USA). The plasmids for His_6-tagged NFATc1 fragments were re-cloned from the NFATc1 plasmid using pfu polymerase-based PCR and blunt-end ligation. The complementary DNA of Luciferase-Trap were cloned by reverse transcription and PCR using Pfu DNA polymerase, and the cDNAs were inserted into pcDNA. NEDD8 cDNA obtained by reverse transcription-PCR was cloned into Flag-tagged pcDNA3. The plasmids of HA-Ubiquitin, His-NEDD8, His-mutant NEDD8 (His-NEDD8ΔGG, which cannot be conjugated with the target protein), Myc-Runx2, Flag-c-Cbl, Flag/streptavidin binding protein(F/S)-tagged Runx2 fragments, along with OG2 reporter plasmids, were constructed. All small interfering RNAs (siRNAs) were synthesized by Integrated DNA Technologies (Coralville, IA) and their sequences are listed in Supplementary Table 3. Plasmids and siRNAs were transiently transfected into cells using Lipofectamine 2000 (Thermo Fisher Scientific, Waltham, MA) and Lipofectamine RNAiMAX (Thermo Fisher Scientific) reagents.

Reporter assay

To examine the transcriptional activity of NFATc1 and Runx2, each protein responsive luciferase reporter plasmids were used. Cells were co-transfected with luciferase reporter plasmid (TRAP-Luc, OG2-Luc), CMV-ß-gal plasmid, His-NEDD8 plasmid or 50 nM siRNA targeting NEDD8 using Lipofectamine 2000 (Thermo Fisher Scientific, Waltham, MA). The final concentration of DNA or siRNA was adjusted with pcDNA and control siRNA. Transfected cells were washed with PBS and then lysed in reporter lysis buffer. Cell lysates were mixed with luciferase assay reagent (Promega Corp.) and luciferase activity was measured in microplate reader SPARK (Tecan, Grödig, Austria). Luciferase activity was normalized with β-galactosidase activity using o-nitrophenyl-β-d-galactopyranoside (Sigma–Aldrich) as a substrate.

Immunoblotting and immunoprecipitation

Protein extractions were separated on SDS-polyacrylamide gels and transferred to Immobilon-P membranes (Millipore, Bedford, MA, USA). Membranes were blocked with 1% skim milk, 1% bovine serum albumin (BSA) or 3% BSA in Tris/saline solution containing 0.1% Tween-20 (TBS-T) for 1 hour and incubated overnight at 4 °C with primary antibody in the respective blocking solution. Membranes were further incubated with a horseradish peroxidase-conjugated secondary antibody (diluted 1:5000 in the respective blocking solution) for 1 h and visualized using the ECL Plus kit (Thermo Fisher Scientific, Waltham,

MA, USA). To precipitate tagged proteins, transfected cells were lysed with buffer containing 50 mM Tris-Cl (pH 8.0), 100 mM NaCl, 5 mM EDTA, 0.1% NP-40, 10% Glycerol and a protease inhibitor cocktail (Sigma-Aldrich). Cell lysates were incubated with EZview anti-Myc or anti-FLAG-M2 affinity beads (Sigma-Aldrich) at 4 °C for 4 h. To precipitate endogenous protein, cell lysates were incubated with antibody at 4 °C overnight and further incubated with Protein A/G Sepharose beads (GE Healthcare Life Sciences, Marlborough, MA, USA) at 4 °C for 4 h. After incubation, the precipitates were washed with lysis buffer three times. The proteins bound to the beads were eluted with SDS denaturing buffer and subjected to western blotting.

Identification of His-NEDD8 conjugates

After transfection of His-NEDD8 or NEDD8-ΔGG mutant, cells were divided into two dishes which lysed with 2x SDS sample buffer and performed Ni^{2+}-NTA-agarose beads assay (Qiagen, Valencia, CA, USA). For Ni^{2+}-NTA-agarose beads assay, sample was lysed with denaturing buffer (6 M guanidine hydrochloride, 0.1 M Na_2HPO_4/NaH_2PO_4, 0.01 M Tris-Cl (pH 8.0), 10 mM Imidazole and 10 mM β-mercaptoethanol). The lysates were incubated with Ni^{2+}-NTA-agarose beads at 4 ℃ for 4 hours. The beads were washed with washing buffer (pH 6.3) plus 0.2% and 0.1% Triton X-100, sequentially. The bound proteins were eluted in a SDS denaturing buffer and subjected to immunoblotting.

Antibodies and reagents

Antibodies against NFATc1 (sc-7294), ubiquitin (sc-8017), Osx (sc-393325) and c-Cbl (sc-170) were obtained from Santa Cruz Biotechnology (Santa cruz, CA, USA). Antibodies against Tubulin (2146S), NEDD8 (2745S), Rbx1 (11922S), p65 (8242S), c-Fos (2250S), JunB (3753S), HA (3724S), and Myc (2276S) were obtained from Cell Signaling Technology (Beverly, MA, USA). β-glycerophosphate, Dexamethasone, Ascorbic acid, Ezview red anti-Flag M2 affinity gel (F2426) and Antibodies against Flag (F7425) were obtained from Sigma-Aldrich (St. Louis, MO, USA). Antibodies against Runx2 (D130-3) and His (D291-3) was obtained from Medical & Biological Laboratories (Tokyo, Japan). The Runx2 (ab236639), NFATc1 (ab2796), and Dlx5 (ab109737) antibodies used for human sample immunohistochemistry were obtained from Abcam (Cambridge, UK). The NAE1 (NBP1-92162) antibody was obtained from Novus Biologicals (Centennial, CO, USA). MG132 (BML-PI102) was obtained from ENZO Life Sciences (Farmingdale, NY, USA). Recombinant Mouse M-CSF (300-25) and Recombinant Mouse BMP2 (120-02C) were obtained from Peprotech (Cranbury, NJ, USA). Ni-NTA agarose beads (30230) were obtained from Qiagen (Hilden, Germany). MLN4924 (HY-70062) was purchased from MedChemExpress (New Jersey, USA). Additional details regarding antibody titers, blocking conditions, and specific applications are summarized in Supplementary Table 4.

RNA extraction, cDNA synthesis and RT-qPCR

Total RNA was extracted from cultured cells using Trizol reagent (Invitrogen, Carlsbad, CA, USA). cDNA was synthesized from 1 μg of total RNAs using an EasyScript cDNA Synthesis Kit (Applied Biological Materials Inc., Richmond, Canada). cDNAs were amplified with EvaGreen qPCR master mix reagent (Applied Biological Materials) using a StepOne Real-time PCR System (Applied Biosystems, Foster City, CA, USA). Their levels were quantified by calculating Ct (threshold cycle) values and normalized by 18S ribosomal RNA (18S rRNA) level as an internal control. The sequences of PCR primers are summarized in Supplementary Table 2.

Chromatin immunoprecipitation (ChIP)

Chromatins were cross-linked with 1% formaldehyde for 15 min, followed by washing with cold PBS. The cells were collected by scraping and centrifugation., and the pellets were lysed in the FA lysis buffer (50 mM HEPES, pH 7.5, 140 mM NaCl, 1 mM EDTA, 1% Triton X-100, 0.1% sodium deoxycholate, 0.1% SDS, and a protease inhibitor cocktail). The lysates were sonicated for 20 sec and 1 min intervals, repeating the process seven times to fragment chromosomal DNAs into 400-700 bp pieces. The lysates were centrifuged and diluted in a chromatin RIPA buffer (50 mM Tris, pH 8.0, 150 mM NaCl, 2 mM EDTA, 1% NP-40, 0.5% sodium deoxycholate, 0.1% SDS, and a protease inhibitor cocktail), with 10% of each sample used as the input control. The samples were immunoprecipitated with 4 μg of anti-Runx2(MBL), NFATc1(CST), and control IgG for 16 h and further incubated with 30 μL of protein A/G-Sepharose beads (GE Healthcare Life Sciences, Marlborough, MA, USA) for 4 h. The samples were washed with a buffer (20 mM Tris, pH 8.0, 0.1% SDS, 1% Triton X-100, 2 mM EDTA, and 150 mM NaCl), and eluted with an elution buffer (1% SDS, 100 mM $NaHCO_3$). Precipitated DNAs and input DNAs were purified and quantified using the ABI StepOne Real-Time PCR System and EvaGreen qPCR Mastermix. PCR primer sequences used for ChIP are listed in Supplementary Table 2.

ELISA

The C-terminal telopeptide of type I collagen (CTX-1) concentration was analyzed using a mouse CTX1 ELISA kit (AC-06F1, Immunodiagnostic System, Liege, Belgium). The procollagen type I N-terminal propeptide (P1NP) concentration was analyzed using a mouse P1NP ELISA kit (E-EL-M0233, Elabscience). After ELISA assays were conducted according to the manufacturer instructions, microplate reader SPARK (Tecan, Grödig, Austria) was used to measure the absorbance (OD) value at 450 nm. According to the OD value of each sample, the concentration of P1NP and CTX1 of the corresponding samples were calculated.

Bioinformatics analysis

Gene expression profile data GSE230665, GSE176265, GSE181459 were collected from the National Center for Biotechnology Information's Gene Expression Omnibus (GEO, NCBI). GSE230665 included 3 healthy postmenopausal women and 12 patients with postmenopausal osteoporosis; GSE176265 included BMMs treated with 30 ng/mL M-CSF and 100 ng/mL RANKL for 0, 24, 48, 72 hours; GSE181459 included primary osteoblasts from 12 wild-type mice and 12 β-Arrestin 2 knockout mice. $|\log2(\text{fold change})| > 1.6$ and P value < 0.05 were used as cut-offs in volcano plot. For examination of gene expression patterns, expression profiles were stratified into two groups using either k-means clustering (implemented in R v.4.4.2) or median-based separation. Two-tailed unpaired *t* tests were used to compute *P* values. Heat maps were generated in R using the Rstudio IDE (Posit Software, PBC, Boston, MA, USA)

Gene set enrichment analysis (GSEA)

Normalized enrichment score (NES), nominal *P* value, and false discovery rate (FDR) were calculated using the Broad Institute GSEA software. Nominal *P* value < 0.01 and FDR < 0.25 were considered significant. Postmenopausal osteoporosis patients were divided into two groups based on the mRNA level of *NAE1*(A_23_P77459; GSE230665) using the upper and lower quartiles to define high NAE1 and low NAE1 groups. Gene sets for "*positive regulation of bone resorption*", "*negative regulation of bone remodeling*", "*negative regulation of osteoblast differentiation*", "*osteoclast differentiation*", and "*positive regulation of osteoblast differentiation*" were obtained from MsigDB (https://software.broadinstitute.org/gsea/msigdb/index.jsp). GSEA was performed using the GSEA software v.4.3.2 (https://software.broadinstitute.org/gsea/downloads.jsp).

Supplementary Figures

Supplementary Fig. 1

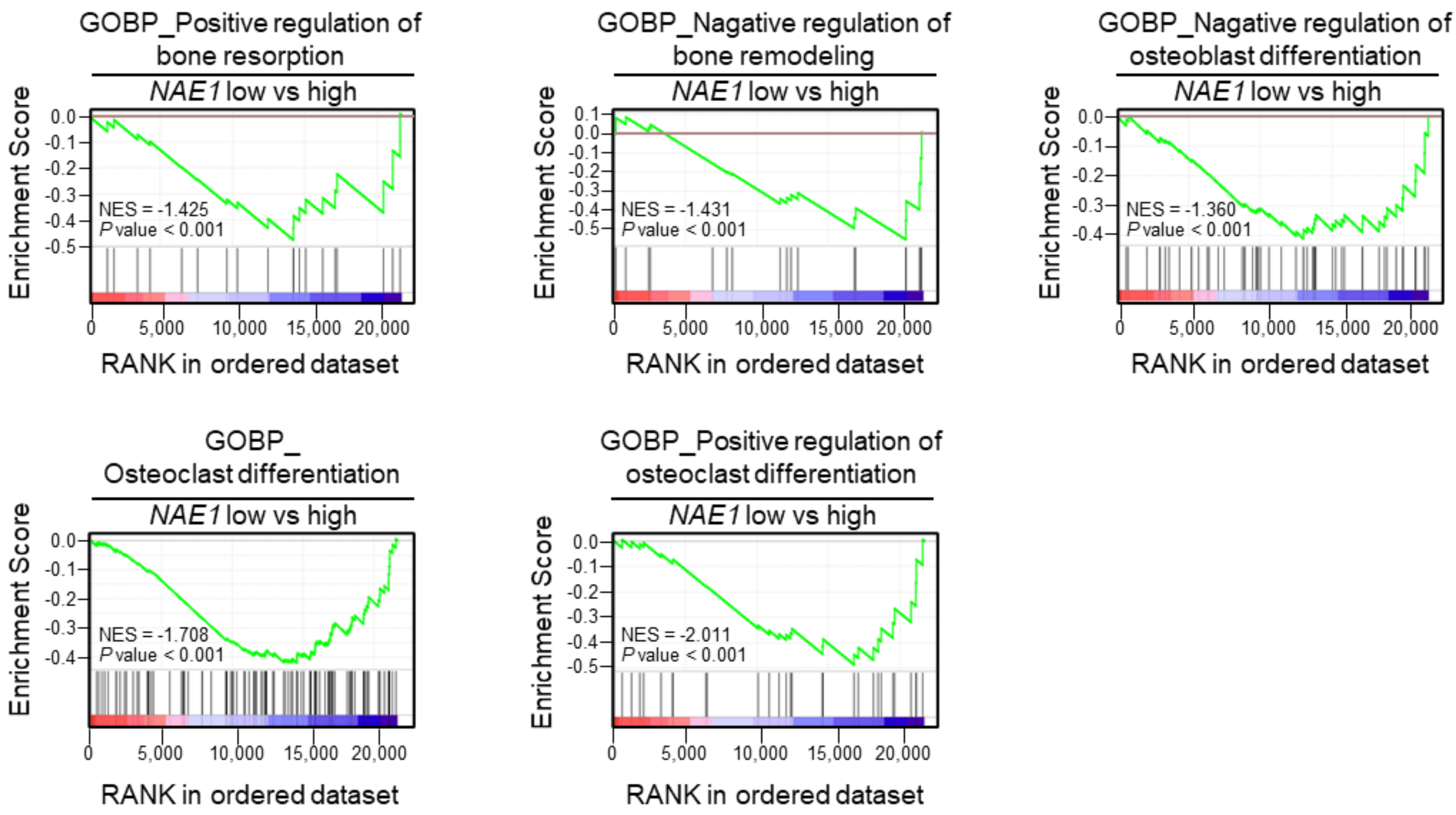

Supplementary Fig. 1: Negative bone remodeling-associated gene sets are enriched in high *NAE1* group in postmenopausal osteoporosis.

Gene enrichment analysis (GSEA) was performed on transcriptome data from GSE230665 PMOP pateints (*n* = 12), stratified into *NAE1*-high and *NAE1*-low groups based on quartile classification. Analysis focused on gene sets related to cellular responses to bone remodeling, including *positive regulation of bone resorption*, *negative regulation of bone remodeling*, *negative regulation of osteoblast differentiation*, *osteoclast differentiation*, and *positive regulation of osteoclast differentiation*. Normalized enrichment scores (NES) and nominal *P* values are shown.

Supplementary Fig. 2

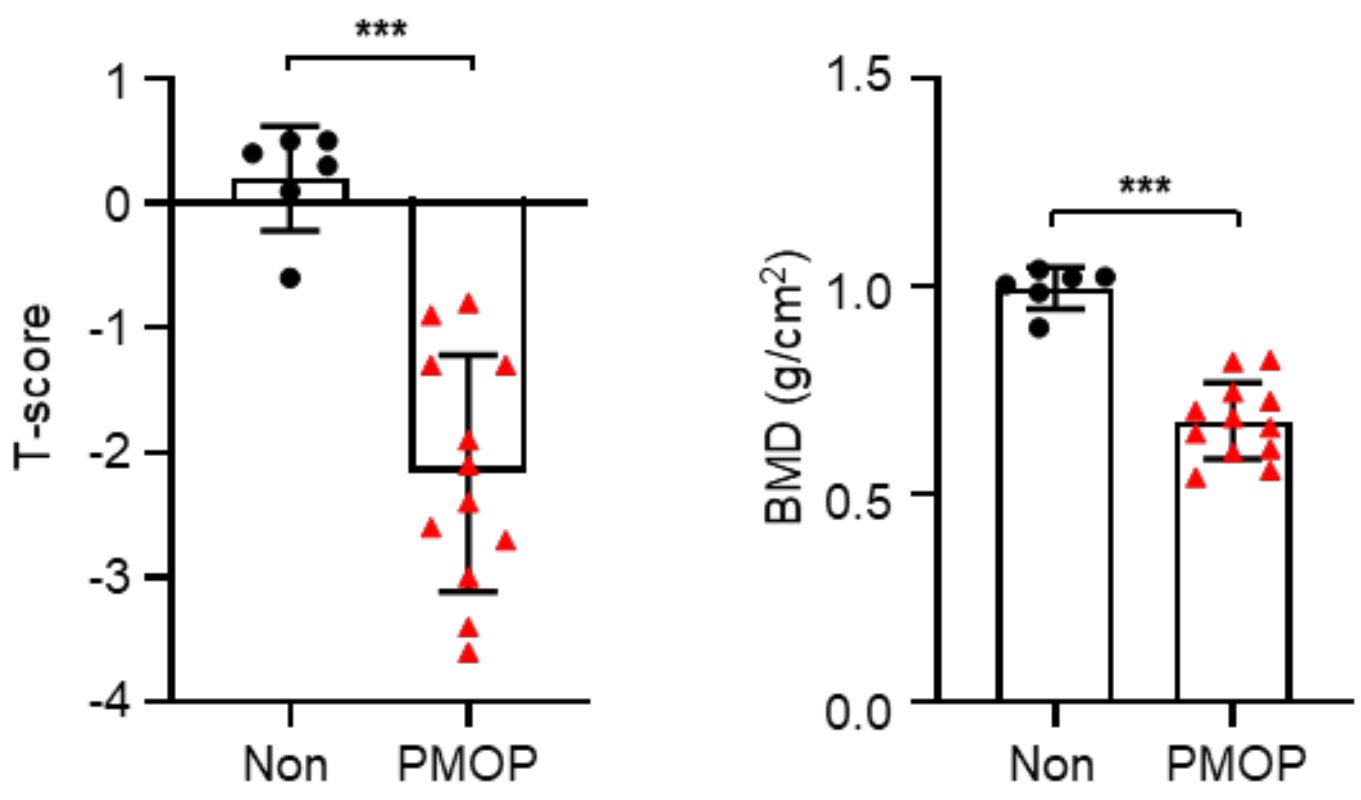

Supplementary Fig. 2: T-score and bone mineral density measurements are compared between non-osteoporotic controls and postmenopausal osteoporotic patients.

T-scores and bone mineral density measurements (BMD) values for non-osteoporotic controls (Non, *n* = 6) and postmenopausal osteoporotic (PMOP, *n* = 12) patients. Measurements were obtained from the left of right proximal femur of each patient using dual-energy X-ray absorptiometry. The data are presented as the mean ± s.d.; ****P* < 0.001. Statistical significance was determined by a two-sided Mann–Whitney *U* test.

Supplementary Fig. 3

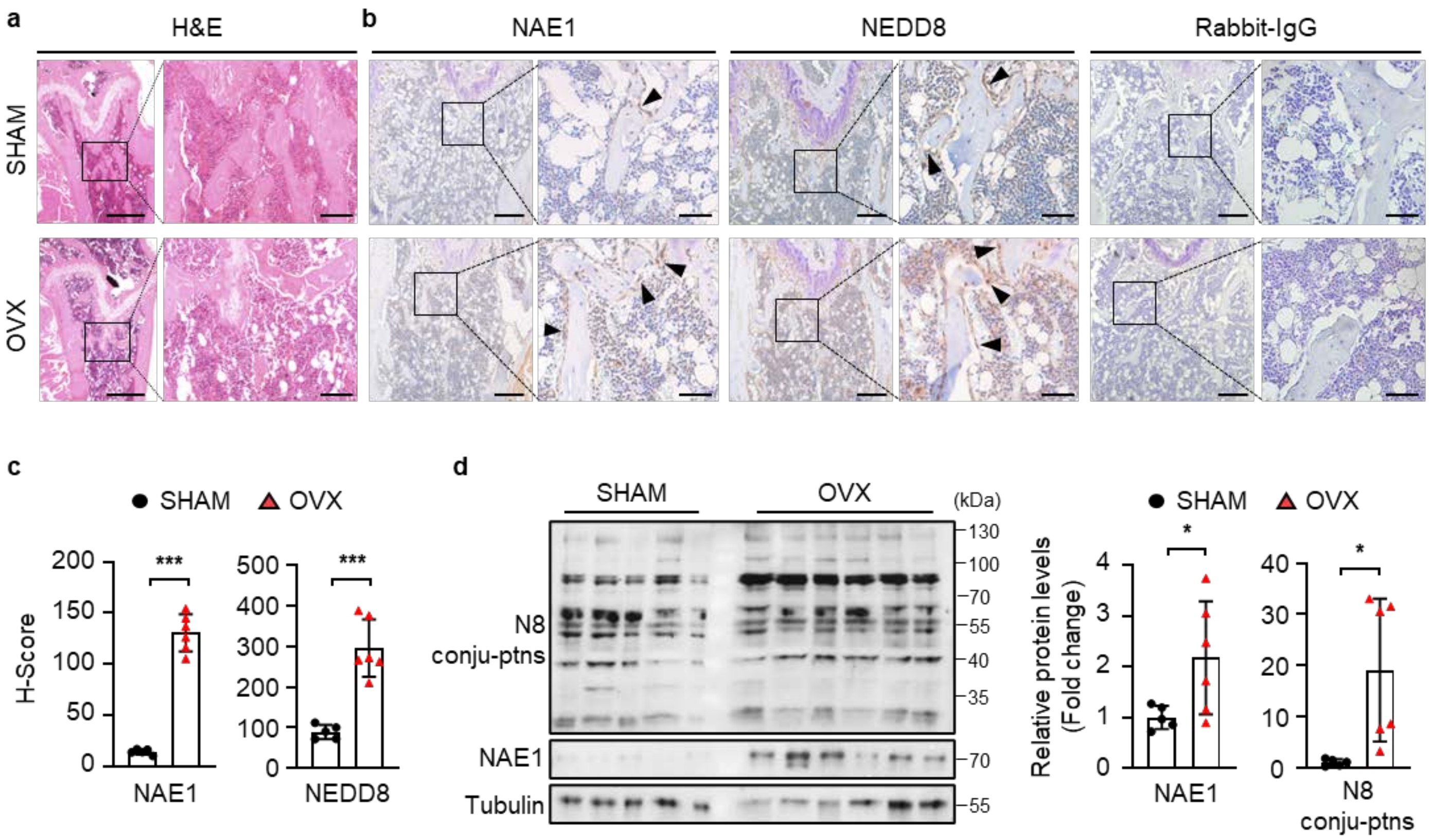

Supplementary Fig. 3: Ovariectomy-induced osteoporosis led to elevated neddylation levels in bone tissue.

a. Representative H&E images of distal femoral section from SHAM (*n* = 5) and ovariectomized (OVX, *n* = 6) groups. Left, low-magnification; right, high-magnification. Scale bars, 500 (left) and 100 μm (right). **b.** Representative immunohistochemistry (IHC) for NAE1, NEDD8, and IgG control in femoral section from SHAM (*n* = 5) and OVX (*n* = 6) groups. Arrowheads indicate positive cells. Left, low-magnification; right, high-magnification. Scale bars, 200 (left) and 50 μm (right). **c.** Quantification of IHC staining by H-score (SHAM, *n* = 5; OVX, *n* = 6). **d.** Immunoblots of NAE1 and NEDD8-conjugated proteins (N8 conju-ptns) in femoral lysates from SHAM and OVX groups. Densitometry was performed using ImageJ, normalized to tubulin (Tub) and expressed relative to the SHAM group. The data are presented as the mean ± s.d. (*n* = 5-6 independent animals in each group); *$P < 0.05$; ***$P < 0.001$. Statistical significance was determined by two-tailed unpaired *t* test.

Supplementary Fig. 4

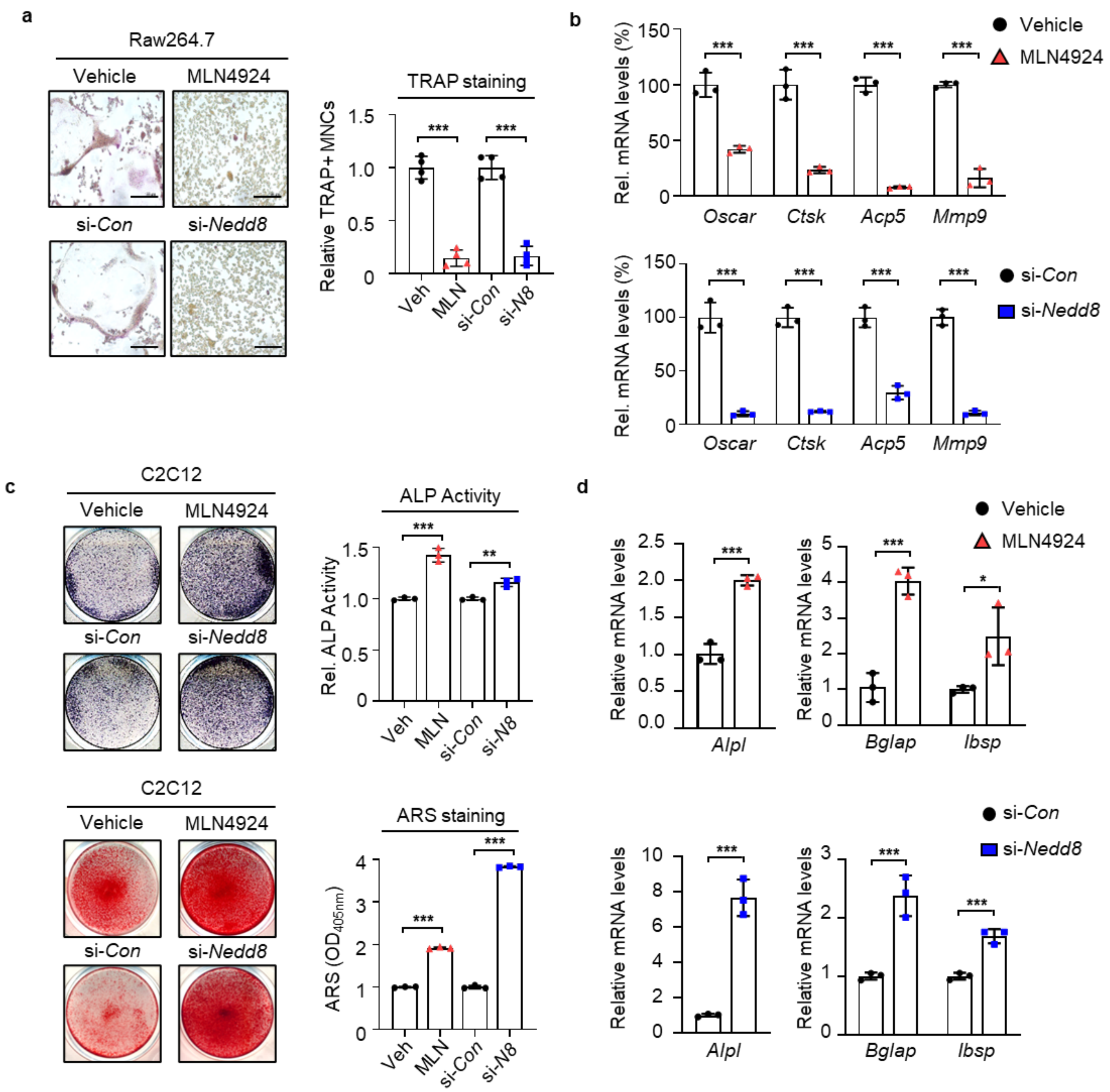

Supplementary Fig. 4: Neddylation inhibition affects osteoclast and osteoblast differentiation in RAW 264.7 and C2C12 cells.

a. Osteoclasts derived from RAW 264.7 cells were treated with either vehicle (Veh), MLN4924 (MLN, 50 nM), control siRNA (si-*Con*) or si-*NEDD8* (si-*N8*) and cultured in α-MEM with RANKL (100 ng/mL) for 3 days. TRAP staining shows TRAP+ multinucleated cells (MNCs; ≥3 nuclei). Scale bars, 100 μm. **b.** Total RNAs were isolated from RAW 264.7 cells treated as in

(**a**). The expression of osteoclast differentiation marker genes was quantified by RT-qPCR. **c.** C2C12 cells were cultured in osteoblast differentiation medium (Ob-DM) and treated with Veh, MLN (50 nM), si-*Con*, or si-*N8*. Representative alkaline phosphatase (ALP) staining (day 3) and Alizarin Red S (ARS) staining (day 5). **d.** Total RNAs were isolated from C2C12 cells treated as in (**c**). The expression of osteoblast marker genes was quantified by RT-qPCR (*Alpl* at day 3, *Bglap* and *Ibsp* at day 5). The data are presented as the mean ± s.d. (**a**, $n = 4$; **b–d**, $n = 3$); *$P < 0.05$; **$P < 0.01$; ***$P < 0.001$. Statistical significance was determined by two-tailed unpaired t test.

Supplementary Fig. 5

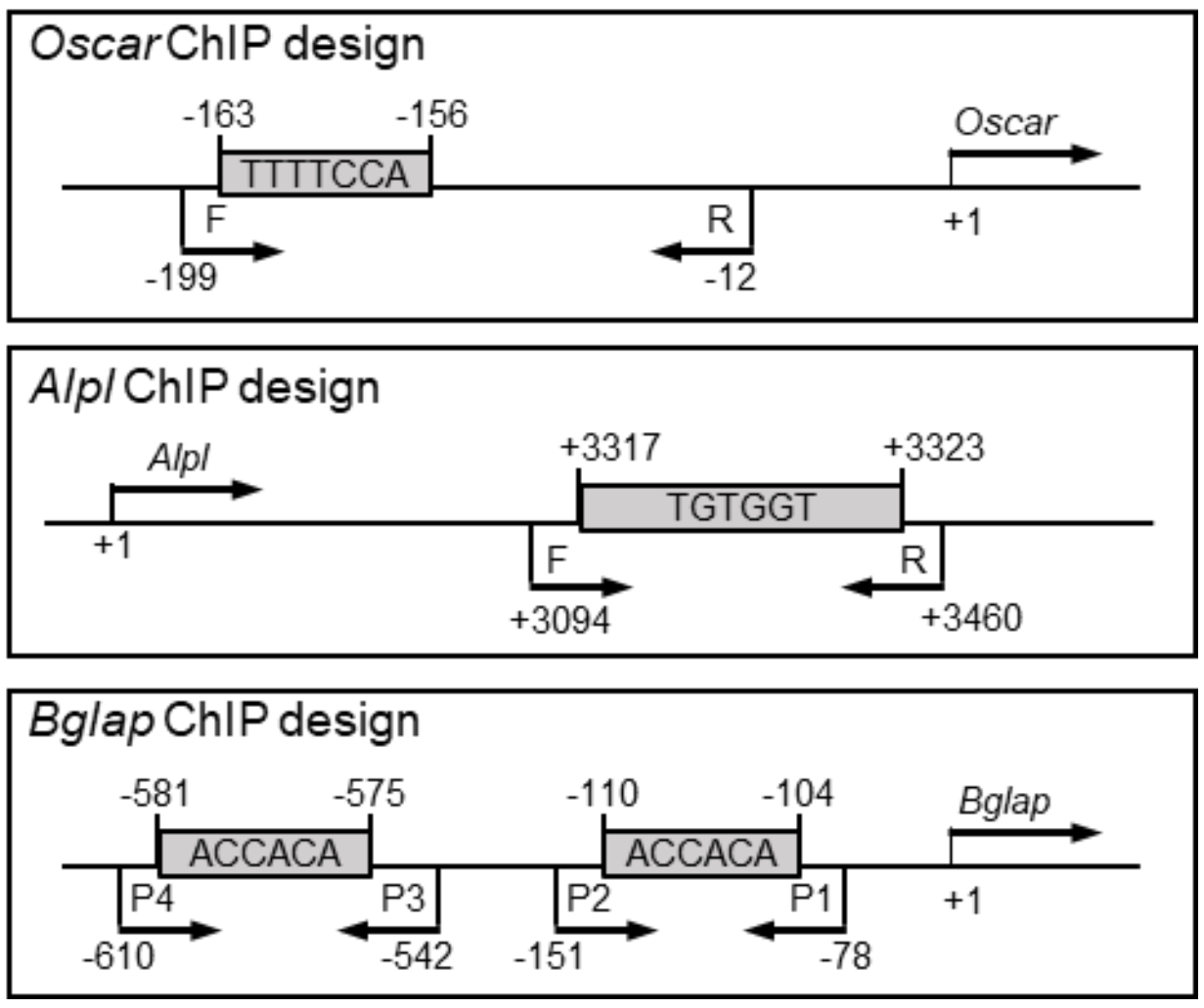

Supplementary Fig. 5: Primer were designed for ChIP-qPCR analysis of transcription factor binding sites.

Schematic representation of primer positions used for ChIP-qPCR assays. Primers were designed to amplify the NFATc1 binding site within the *Oscar* promoter, the Runx2 binding site within the *Alpl* enhancer, and the *Bglap* promoter.

Supplementary Fig. 6

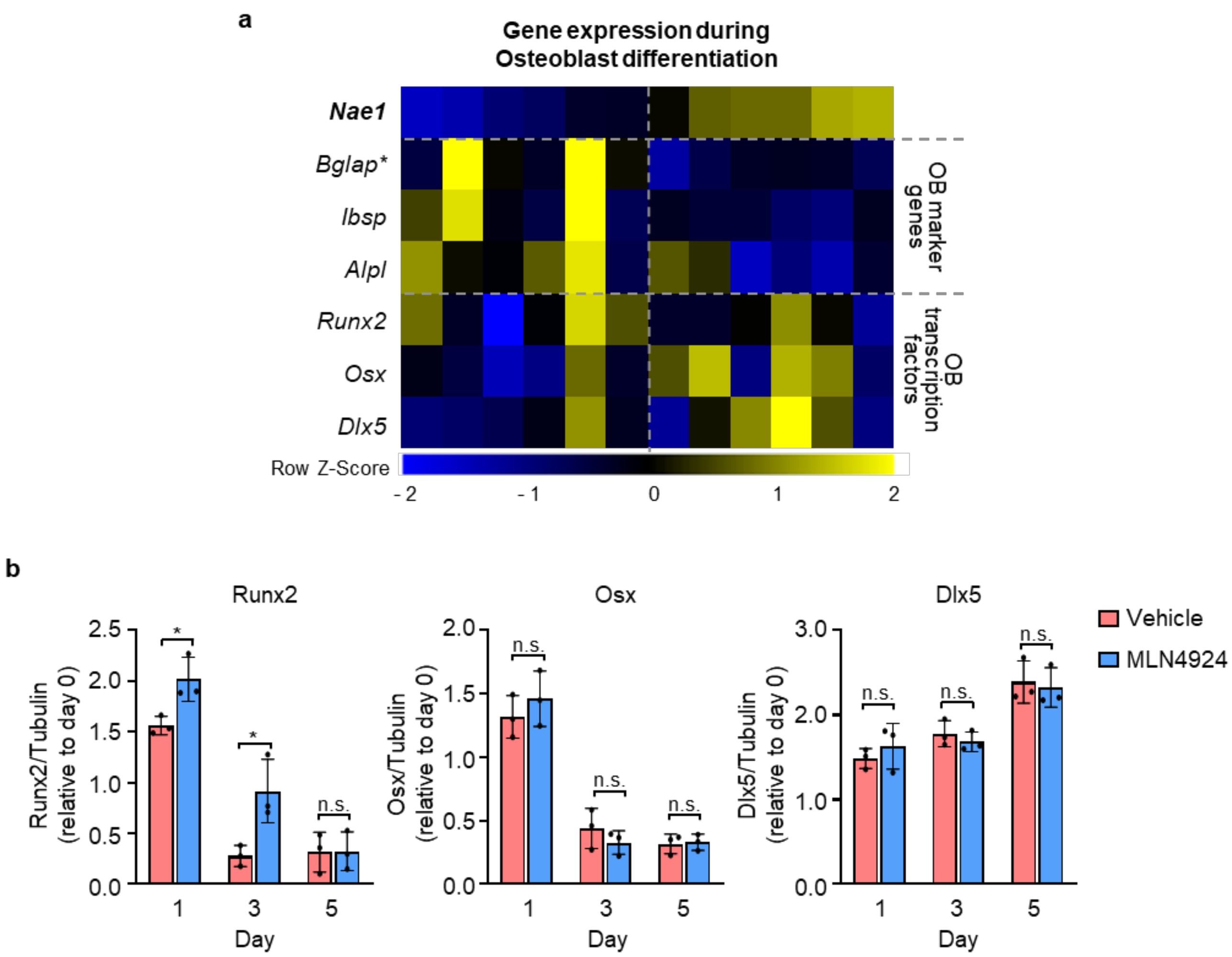

Supplementary Fig. 6: Heat map shows transcription factors and osteoblast marker genes stratified by *Nae1* expression.

a. Heat map of GSE181459 showing expression patterns during osteoblast (OB) differentiation. Samples were stratified by median split of *Nae1* expression. The panel displays OB marker genes and OB-related transcription factors. Asterisks indicate genes significantly different between groups (*$P < 0.05$). The color scale bar represents row Z-scores, ranging from low (blue) to high (yellow). **b.** Quantification of Runx2, Osx, and Dlx5 protein levels in the immunoblots shown in Fig. 5a. Protein levels at each time point were quantified by immunoblot densitometry and expressed relative to day 0. The data are presented as the mean ± s.d. (n = 3); n.s., not significant; *$P < 0.05$; **$P < 0.01$; ***$P < 0.001$. Statistical significance was determined by two-tailed unpaired *t* tests.

Supplementary Fig. 7

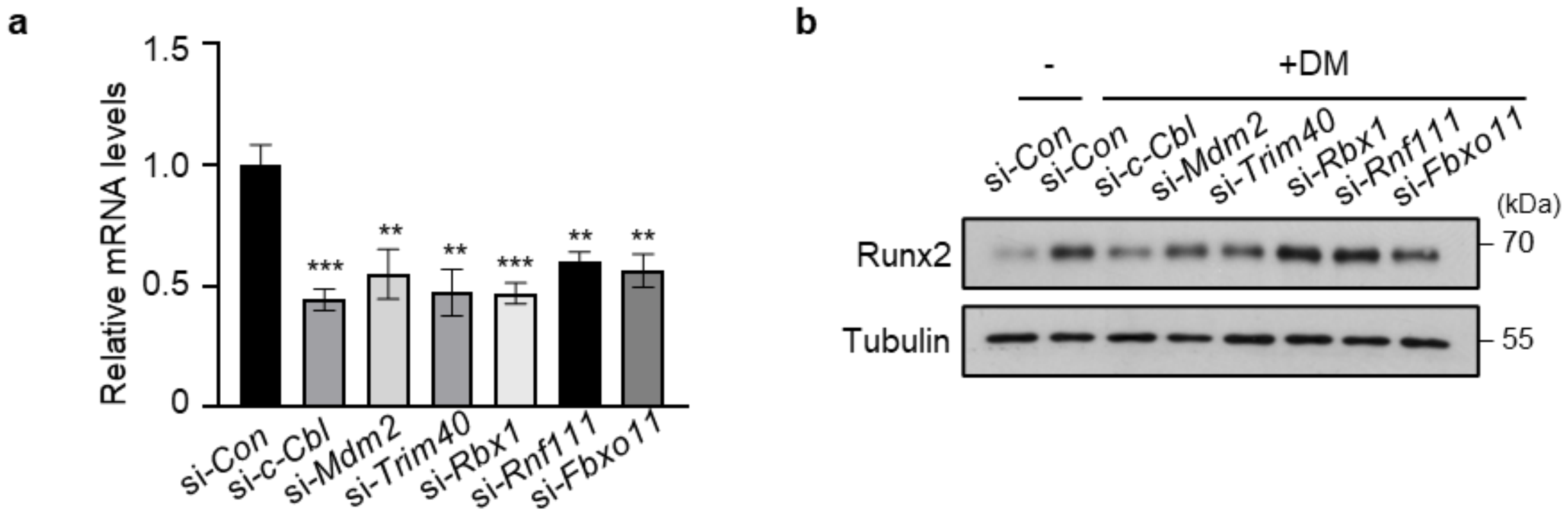

Supplementary Fig. 7: Screening identifies neddylation E3 ligase candidate responsible for Runx2 in osteoblast differentiation.

a. C2C12 cells were transfected with siRNAs targeting *c-Cbl, Mdm2*, *Trim40, Rbx1*, *Rnf111*, or *Fbxo11* (si-*Con* as control). Relative mRNA levels of the target genes were quantified by RT-qPCR. The data are presented as mean ± s.d. (n = 3 independent experiments); **P < 0.01; ***P < 0.001 versus si-*Con*. Statistical significance was determined by two-tailed unpaired t test. **b.** Immunoblot of Runx2 in C2C12 cells transfected with the indicated siRNAs and cultured in Ob-DM for 1 day (n = 3 independent experiments). These immunoblots serve as the source for the quantification shown in Fig. 6b.

Supplementary Fig. 8

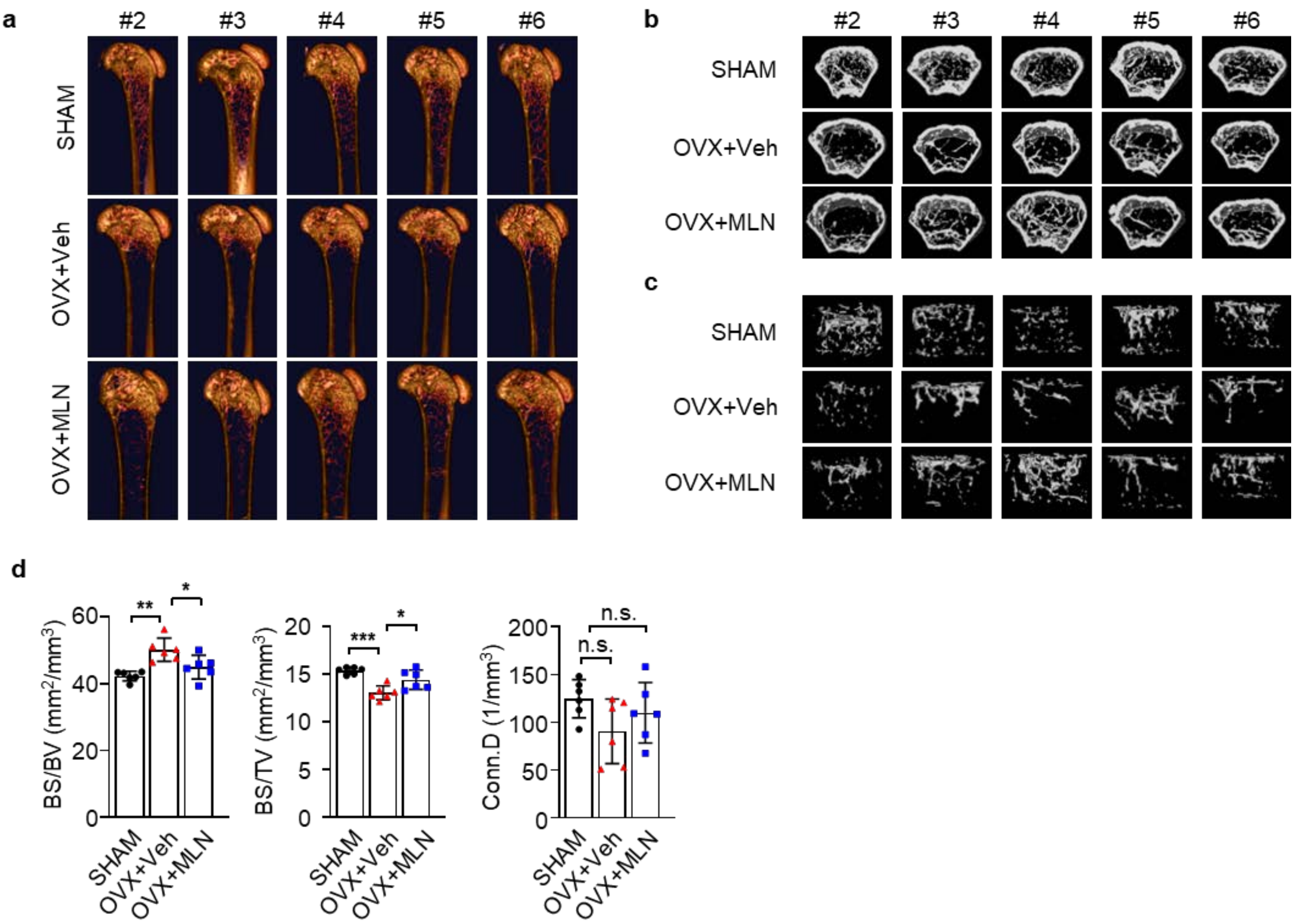

Supplementary Fig. 8: Inhibition of neddylation by MLN4924 attenuates bone loss in osteoporotic mice.

Panels **a–d** were obtained from the same in vivo cohorts as Fig. 7 (n = 6 mice per group). **a.** Longitudinal micro-computed tomography (micro-CT) images of distal femora from SHAM, OVX+Veh, and OVX+MLN groups. **b.** Cross-sectional micro-CT images of the distal metaphyseal region from SHAM, OVX+Veh, and OVX+MLN groups. **c.** 3D-reconstrcuted micro-CT images of the distal metaphyseal region from SHAM, OVX+Veh, and OVX+MLN groups. **d.** Quantitative micro-CT analysis of distal femora: bone surface/bone volume (BS/BV), bone surface/total volume (BS/TV), and connectivity density (Conn.D). The data are presented as the mean ± s.d.; n.s., not significant; *P < 0.05; **P < 0.01; ***P < 0.001. Statistical significance was determined by one-way ANOVA followed by Bonferroni's post hoc test.

Supplementary Fig. 9

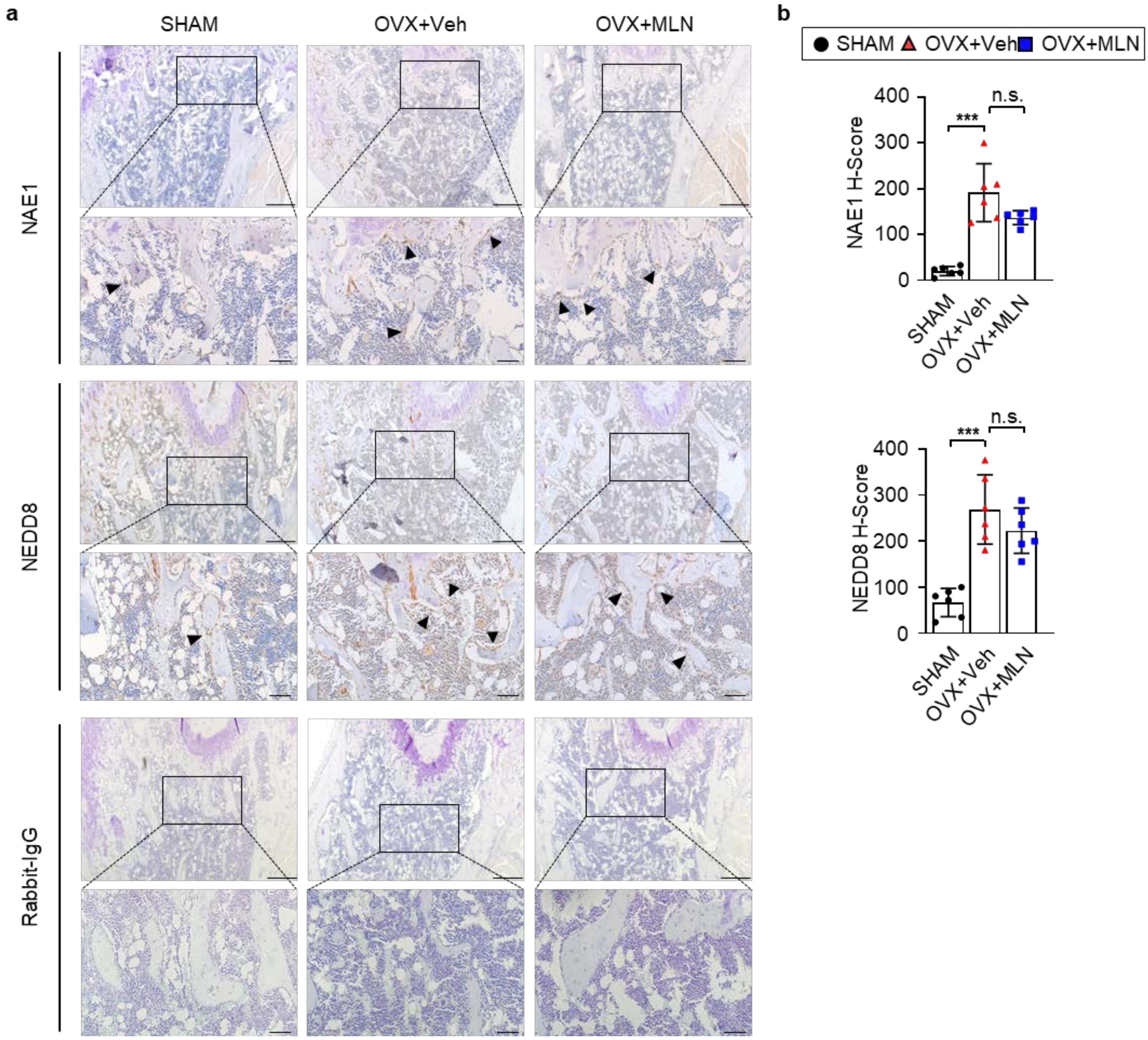

Supplementary Fig. 9: The expression of NAE1 and NEDD8 is shown in bone tissues.

a. Representative immunohistochemistry for NAE1, NEDD8, and IgG control in distal femoral sections from SHAM, OVX, and OVX+MLN groups (*n* = 6 independent animals in each group). Arrowheads indicate positive cells. Top, low-magnification; bottom, high-magnification. Scale bar, 200 (top) and 50 µm (bottom). **b.** Quantification of IHC staining by H-score. The data are presented as mean ± s.d.; n.s., not significant; ***$P < 0.001$. Statistical significance was determined by one-way ANOVA followed by Bonferroni's post hoc test.

Supplementary Fig. 10

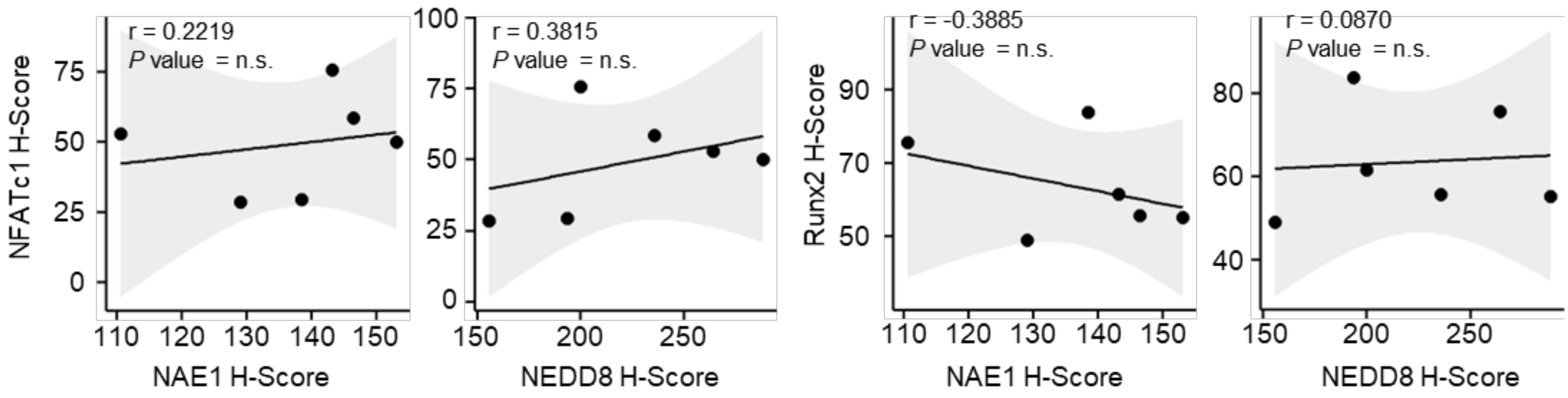

Supplementary Fig. 10: MLN4924 treatment attenuates correlations between neddylation and NFATc1/Runx2 expression.

Correlations between H-scores of NAE1 or NEDD8 and NFATc1 (left) or Runx2 (right) in the OVX+MLN group (*n* = 6) from the OVX mouse experiment shown in Fig. 7. Pearson's correlation coefficient (r) and *P* values were determined by linear regression; shaded area, 95% confidence interval.

Supplementary Fig. 11

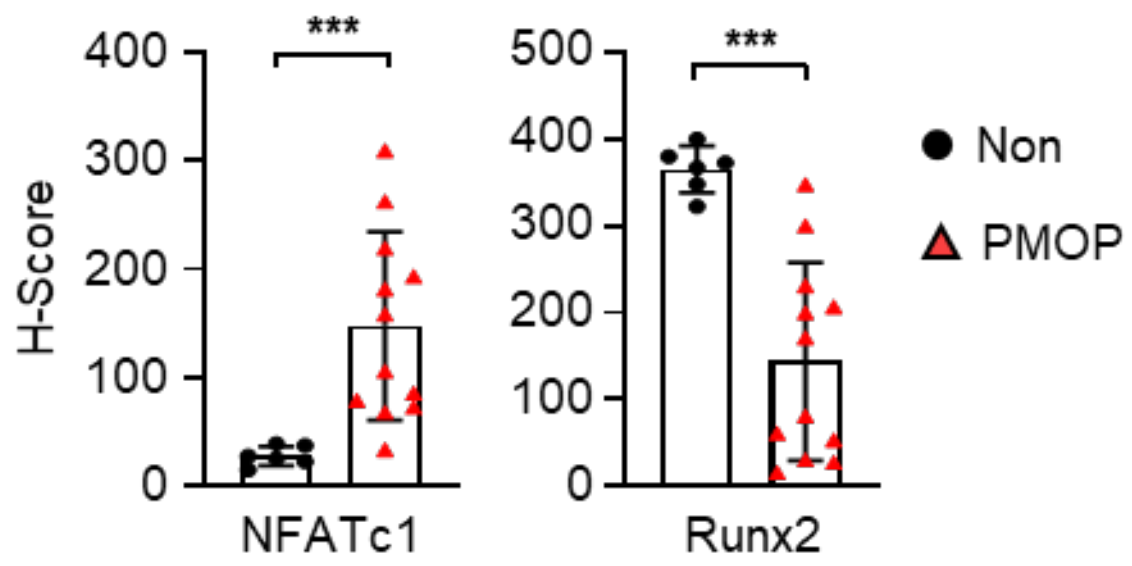

Supplementary Fig. 11: Quantification of NFATc1 and Runx2 expression in femoral head sections from human samples.

Staining intensity in Fig. 8b was quantified by H-score. The data are presented as mean ± s.d. (Non, *n* = 6; PMOP, *n* = 12); ***$P < 0.001$. Statistical significance was determined by two-sided Mann–Whitney *U* test.

Supplementary Tables

Supplementary Table 1. Patient information used in this study.

Sex: Female (100%)		Normal (*n* = 6)	Postmenopausal osteoporosis (*n* = 12)	*P* value
Age (years)		66 (47.1 to 73.5)	72 (67.7 to 82.2)	0.0213
BMD (g/cm^2)	Proximal Femur	1.013 (0.990 to 1.022)	0.673 (0.607 to 0.729)	0.0001
	Lumbar Spine (L1-L4)	1.170 (1.153 to 1.254)	0.844 (0.805 to 0.915)	$P < 0.0001$
T-SCORE	Proximal Femur	0.35 (0.15 to 0.475)	-2.25 (-2.775 to -1.30)	0.0001
	Lumbar Spine (L1-L4)	-0.05 (-0.10 to 0.83)	-2.55 (-2.85 to -1.98)	$P < 0.0001$

Values are presented as median (IQR). Statistical significance was determined by two-sided Mann–Whitney *U* test. BMD, bone mineral density; PMOP, postmenopausal osteoporosis.

Supplementary Table 2. Nucleotide sequences of primers used in experiments.

	Genes	Species	Forward Primers (5' to 3')	Reverse Primers (5' to 3')
RT-qPCR	*Nfatc1*	*Mus musculus*	CCCGTCACATTCTGGTCCAT	CAAGTAACCGTGTAGCTGCACAA
	Oscar	*Mus musculus*	CCCCTCTGCAGTATATTGAC	TATAGTCCAAGGAGCCAGAA
	Ctsk	*Mus musculus*	GACATGACCAGTGAAGAAGT	TGACTGGAGTAACGTATCCT
	Acp5	*Mus musculus*	CAGCTCCCTAGAAGATGGATTCAT	GTCAGGAGTGGGAGCCATATG
	Mmp9	*Mus musculus*	CATGCACTGGGCTTAGATCA	TGTGGTTCAGTTGTGGTGGT
	Runx2	*Mus musculus*	TCCACCACGCCGCTGTCTT	TTGGGAACTGCCTGGGGTCT
	Alpl	*Mus musculus*	CCAACTCTTTTGTGCCAGAGA	GGCTACATTGGTGTTGAGCTTTT
	Bglap	*Mus musculus*	GCAATAAGGTAGTGAACAGACTCC	GTTTGTAGGCGGTCTTCAAGC
	Ibsp	*Mus musculus*	AAGCAGCACCGTTGAGTATGG	CCTTGTAGTAGCTGTATTCGTCCTC
	Cbl	*Mus musculus*	AGCTCCTGAAACCCTGTCTC	CATCGTATGTGTGCCTGGTG
	Mdm2	*Mus musculus*	TACAGCCTGAGTGACGAAGG	AGTTCTCACGAAGGGTCCAG
	Trim40	*Mus musculus*	CTATCTGCCTGGATCCCCTG	AAGAGATGTCCGCTAGCCTC
	Rbx1	*Mus musculus*	CACGAGGAACCTGTCAGAGT	TGATGCCAGTCACTCGTTCT
	Rnf111	*Mus musculus*	TGACGTGAGGAGAACTGCAT	ATGGTGGTGATGGTGGTGAT
	Fbxo11	*Mus musculus*	CGGGCAGACTGGAGGAATAT	TATAAGGCCTCGTCCGTCAC
	Rn18s	*Mus musculus*	TTCGTATTGAGCCGCTAGA	CTTTCGCTCTGGTCCGTCTT
ChIP-qPCR	*Alpl* intronic enhencer	*Mus musculus*	CCCAGCCTCGCTGTGGCATG	ACTGGCCAGGCTCAGACCCT
	Proximal *Bglap* promoter	*Mus musculus*	CGTCCACTCCCAGAGCCTT (P2)	CTCAGTGGGTCAAACCCAAAG (P1)
	Distal *Bglap* promoter	*Mus musculus*	CCCACAATGGGCTAGGCTC (P4)	TGCCTCCATAAGATCCGGTT (P3)
	Oscar promoter	*Mus musculus*	GAACACCAGAGGCTATGACTGTTC	CCGTGGAGCTGAGGAAAAGGTTG

Supplementary Table 3. Nucleotide sequences of siRNAs used in experiments.

	**Genes**	**Species**	**Sequences (5' to 3')**
**siRNAs**	*Nedd8 #1*	*Mus musculus*	CAGACAAGGUGGAGCGAAUCAAGGA
	Nedd8 #2	*Mus musculus*	GGAAGAAGAUGCUAAUUAAAGCGAA
	NEDD8 #1	*Homo sapiens*	AGCGGUAGGAGCAGCAAUUUAUCCG
	Cbl #1	*Mus musculus*	GAGAAUCAACUCAGAACGAAAAGCC
	Cbl #2	*Mus musculus*	GAGAAUCAACUCAGAACGAAAAGCC
	CBL #1	*Homo sapiens*	GAAGUUACCUAAUAAUCCAAAGATG
	CBL #2	*Homo sapiens*	GUUUCCUGAUGGACGAAAUCAGAAT
	Fbxo11	*Mus musculus*	GGUAUUGAAAUUACAAAUCAUGCAA
	Rbx1	*Mus musculus*	GACAUGCAGCAAUCUCUAGAACAAA
	Trim40	*Mus musculus*	UAGCUCUUCUGACUCUGAUAGAACC
	Rnf111	*Mus musculus*	GUCAACCAAAGAUGGCAUGACUUAC
	Mdm2	*Mus musculus*	GCAAUGAUCUACAGAAAUUUAGUGG
	Runx2 #1	*Mus musculus*	ACCGUCAUGGCCGGGAAUGAUGAGA
	Runx2 #2	*Mus musculus*	GAAUAAAAUUCCAUGUCUUUUGATA
	Nfatc1 #1	*Mus musculus*	CCAAGUCAGUUUCUAUGUCUGCAAC
	Nfatc1 #2	*Mus musculus*	AAGCUCAGAAACUCUGAUAUUGAGC
	Control		UUGAGCAAUUCACGUUCAUTT

Supplementary Table 4. The list of antibodies for immunoblotting, immunofluorescence and immunohistochemistry

Antibodies	Source	Identifier	Blocking buffer	Dilution
NFATc1	Santa Cruz Biotechnology	sc-7294	1%BSA + 1% skim milk (IB)<br>3%BSA (IF, IHC)	1:1,000 (IB)<br>1:150 (IF, IHC)
Ubiquitin	Santa Cruz Biotechnology	sc-8017	1%BSA + 1% skim milk (IB)	1:2,000 (IB)
c-Cbl	Santa Cruz Biotechnology	sc-170	1%BSA + 1% skim milk (IB)	1:1,000 (IB)
Osx	Santa Cruz Biotechnology	sc-393325	1%BSA + 1% skim milk (IB)	1:1,000 (IB)
Tubulin	Cell Signaling Technology	2146S	1%BSA + 1% skim milk (IB)	1:2,000 (IB)
NEDD8	Cell Signaling Technology	2745S	3%BSA (IB)<br>3%BSA (IHC)	1:500 (IB)<br>1:200 (IHC)
Rbx1	Cell Signaling Technology	11922S	1%BSA + 1% skim milk (IB)	1:1,000 (IB)
p65	Cell Signaling Technology	8242S	1%BSA + 1% skim milk (IB)	1:1,000 (IB)
c-Fos	Cell Signaling Technology	2250S	1%BSA + 1% skim milk (IB)	1:1,000 (IB)
JunB	Cell Signaling Technology	3753S	1%BSA + 1% skim milk (IB)	1:1,000 (IB)
HA	Cell Signaling Technology	3724S	3%BSA (IB)	1:1,000 (IB)
MYC	Cell Signaling Technology	2276S	1%BSA + 1% skim milk (IB)	1:1,000 (IB)
Flag	Sigma-Aldrich	F7425	1%BSA + 1% skim milk (IB)	1:1,000 (IB)
Runx2	Medical & Biological Laboratories	D130-3	1%BSA + 1% skim milk (IB)<br>3%BSA (IHC)	1:1,000 (IB)<br>1:150 (IHC)
His	Medical & Biological Laboratories	D291-3	1%BSA + 1% skim milk (IB)	1:1,000 (IB)
NAE1	NOVUS Biologicals	NBP1-92162	3%BSA (IB)<br>3%BSA (IHC)	1:1,000 (IB)<br>1:200 (IHC)
Runx2	Abcam	ab236639	3%BSA (IF, IHC)	1:200 (IF, IHC)
NFATc1	Abcam	ab2796	3%BSA (IF, IHC)	1:200 (IF, IHC)
Dlx5	Abcam	ab109737	1%BSA + 1% skim milk (IB)	1:1,000 (IB)

* IB: Immunoblotting, IHC: Immunohistochemistry, IF: Immunofluorescence
